# The Effect of a Single Trifluoromethyl Substituent on the Reactivity of Chelating *C*_2_ and *C*_s_‑Symmetric Bis(alkoxide) Ligands on a Terphenyl Platform

**DOI:** 10.1021/acs.inorgchem.5c05470

**Published:** 2026-01-15

**Authors:** Ruwandhi Jayasundara, Lakshani W. Kulathungage, Benjamin J. Baillie, Cassandra L. Ward, Richard L. Lord, Stanislav Groysman

**Affiliations:** ∇ Department of Chemistry, 2954 Wayne State University, 5101 Cass Ave., Detroit, Michigan 48202, United States; ‡ Lumigen Instrument Center, Wayne State University, 5101 Cass Avenue, Detroit, Michigan 48202, United States; § Department of Chemistry, 1142 Grand Valley State University, 1 Campus Dr, Allendale, Michigan 49401, United States

## Abstract

Herein we describe the synthesis and preliminary reactivity studies of new racemic *C*_2_-symmetric and meso *C*_s_-symmetric bis­(alkoxide) ligands on a *para*-terphenyl platform. The ligands were synthesized by reaction of 2,2′-dilithium-*p*-terphenyl with trifluoroacetophenone, separated by column chromatography, and obtained in 36% (racemic, *rac*-Lig^2^H_2_) and 26% (meso, *meso*-Lig^2^H_2_) isolated yields. The reaction with *n*-butyl-*sec*-butylmagnesium led to formation of the expected *C*_2_-symmetric and *C*_s_-symmetric mononuclear magnesium complexes. In contrast, the reaction with Cr­(N­(SiMe_3_)_2_)_2_(THF)_2_ exhibited a profoundly different coordination chemistry from that of all-phenyl chelating bis­(alkoxide) or monodentate alkoxides. While the latter generally form Cr_2_(OR)_4_ dimers in which the geometry at Cr­(II) is Y-shaped, these new ligands lead to square-planar Cr­(II) complexes. DFT calculations help rationalize the distinct dimeric species Cr_2_(*rac*-Lig^2^)_2_(THF)_4_ and Cr_2_(*meso*-Lig^2^)_2_ observed for these new ligands.

## Graphical abstract

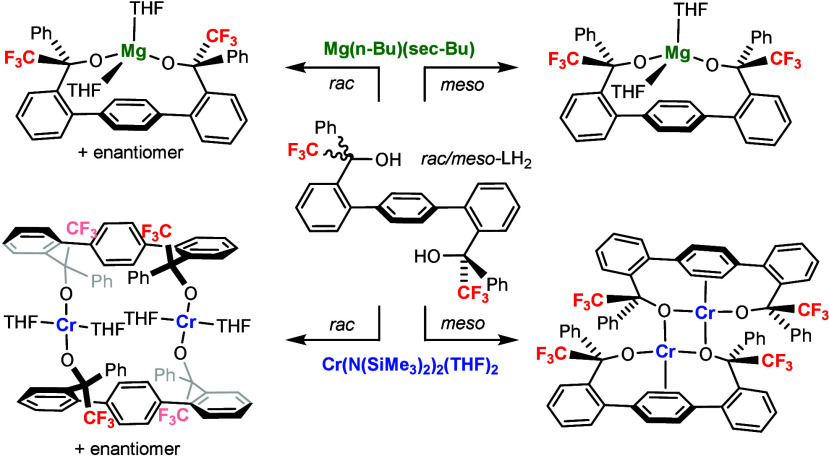

*C*_2_-symmetric bis­(alkoxide) ligands are among the most “privileged” ligands in catalysis, providing support for a variety of *C*_2_-symmetric transition metal or main group catalysts capable of stereoselective transformations.^1,2^ Among other examples, chiral *C*_2_-symmetric TADDOL,^3,4^ BINOL,^5,6^ and SALEN^7,8^ ligands are well-known platforms for asymmetric catalysis. Furthermore, racemic (or achiral) bis­(phenoxide)/bis­(alkoxide) ligands (e.g. SALAN) can lead to *C*_2_-symmetric complex catalysts capable of stereotactic polymerization or other stereoselective applications.^7–27^ However, many of these ligands lack steric bulk, which can render the resulting *C*_2_-symmetric complexes coordinatively saturated and therefore less reactive. In contrast, bulky alkoxide ligands are well-known to lead to low-coordinate reactive metal centers.^28–31^ We described the chemistry of bulky monodentate alkoxide ligands ([OC^*t*^Bu_2_Ph] and [OC^*t*^Bu_2_(3,5-Ph_2_C_6_H_3_)]) with middle and late transition and main-group metals.^32–38^ Due to their steric bulk, they typically result in bis­(alkoxide) mononuclear low-coordinate reactive metal centers that exhibit group-transfer chemistry. While [M­(OR)_2_] platforms exhibited promising catalytic reactivity in the formation of aziridines, cyclopropanes, and azoarenes, they also exhibited some notable limitations pertaining to catalytic performance, including alkoxide lability and the lack of stereoselective catalysis. To minimize alkoxide lability, we pursued a chelating bis­(alkoxide) ligand on a terphenyl platform Lig^1^H_2_ (Figure 1, left).^39^ However, this *C*_2v_-symmetric ligand lacked stereocenters, which therefore cannot potentially lead to stereoselective catalysis. Herein, we report the synthesis and coordination chemistry of a new structurally related *C*_2_-symmetric ligand *rac*-Lig^2^H_2_ and its heterochiral counterpart *meso*-Lig^2^H_2_.

**Figure 1:**
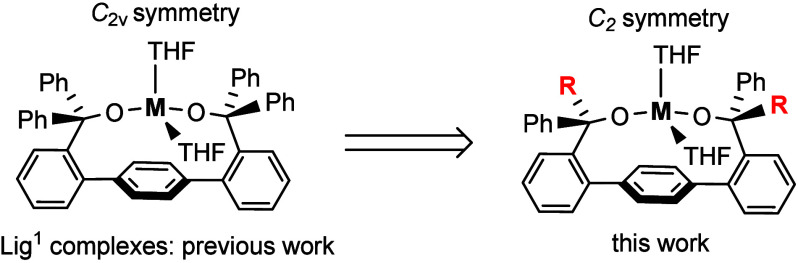
Previous design of *C*_2v_-symmetric complexes with chelating bis­(alkoxide) on a terphenyl platform (Lig_1_), and current design of *C*_2_-symmetric chelating bis­(alkoxide).

Synthesis of the *C*_2v_ ligand Lig^1^H_2_ relied on a three-step procedure that involved preparation of 1,4-bis­(2-bromophenyl)­benzene,^40–42^ lithium to halogen exchange with ^*t*^BuLi, and finally reaction with benzophenone. En route to the *C*_2_-symmetric ligand, we substituted benzophenone with acetophenone initially, but this reaction failed to produce the desired ligand. Identification of *para*-terphenyl as the major product suggested that the relatively acidic protons of acetophenone are incompatible with a strong base. We hypothesized that replacement of acetophenone CH_3_C­(O)­Ph with trifluoroacetophenone CF_3_C­(O)­Ph would enable formation of the desired product by eliminating the acidic protons. Gratifyingly, this reaction led to formation of Lig^2^H_2_ (Figure 2). The product was obtained as a mixture of two diastereomers: the *C*_s_-symmetric heterochiral (*meso*) diastereomer and the *C*_2_-symmetric racemic homochiral diastereomer. The diastereomers were separated by column chromatography to produce homochiral *rac*-Lig^2^H_2_ and *meso*-Lig^2^H_2_ in 36% yield and 26% isolated yield, respectively.

**Figure 2:**
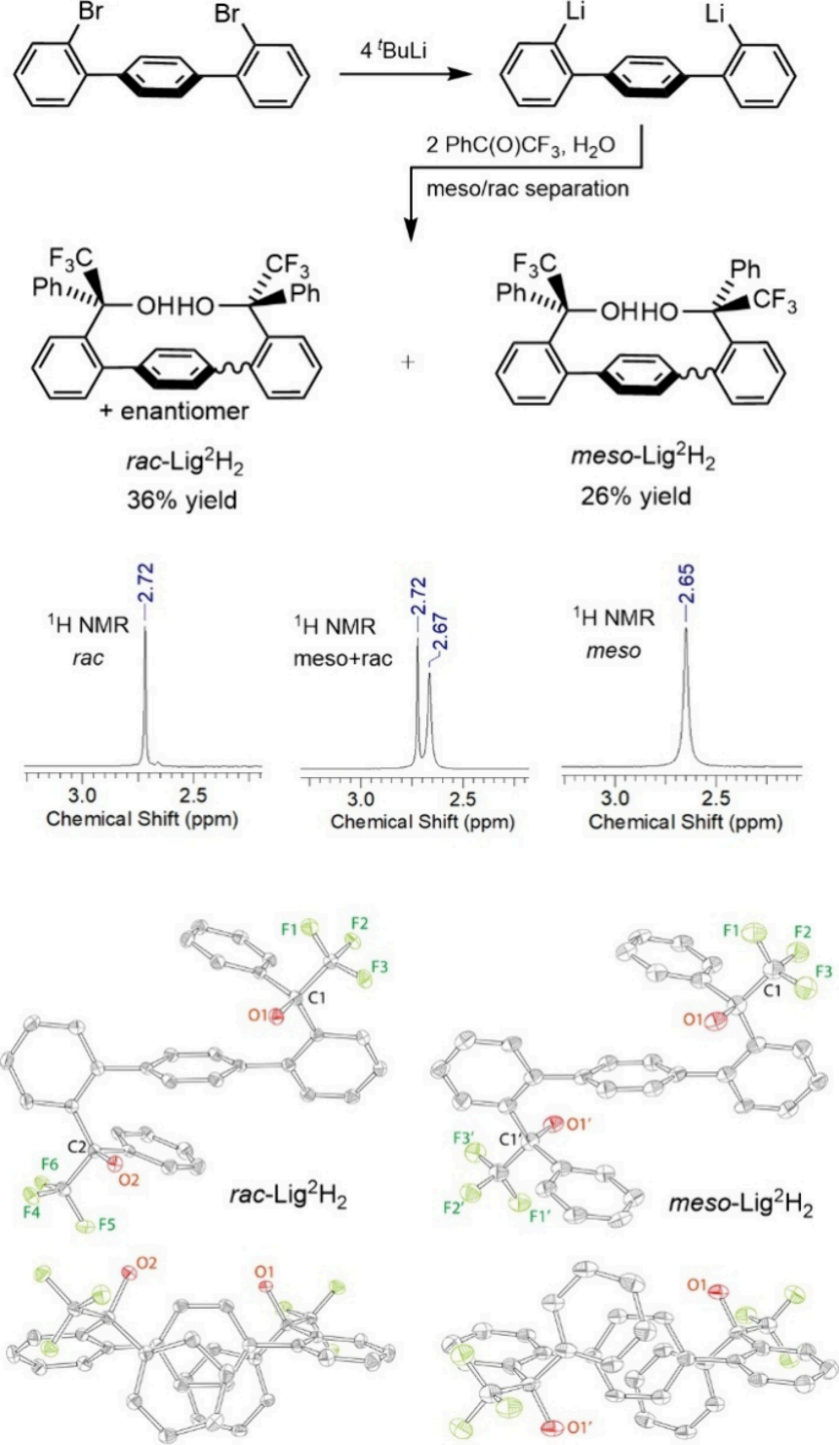
Top: Synthesis and isolated yields of Lig^2^H_2_. Middle: ^1^H NMR of the OH protons of the mixture of diastereomers (center), separated *racemic* (homochiral, left) and *meso* (right) diastereomers. Bottom: X-ray structures (ORTEP drawings, 50% probability ellipsoids, side and top view) of *rac*-Lig^2^H_2_ and *meso*-Lig^2^H_2_.

Both *rac*-Lig^2^H_2_ and *meso*-Lig^2^H_2_ were characterized by ^1^H, ^13^C, and ^19^F NMR, high-resolution mass spectrometry, and X-ray crystallography. The solid-state structures of both ligands are given in Figure 2. The structures confirm the homochiral (RR/SS) nature of the *rac*-Lig^2^H_2_ ligand and the heterochiral RS nature of the *meso*-Lig^2^H_2_ ligand. *Rac*-Lig^2^H_2_ crystallizes in the centrosymmetric *P*-1 space group; only one enantiomer is shown. The whole molecule of *rac*-Lig^2^H_2_ of approximate (noncrystallographic) *C*_2_-symmetry occupies an asymmetric unit. In contrast, *meso*-Lig^2^H_2_ exhibits crystallographic *C*_i_-symmetry, with only half of the ligand constituting an asymmetric unit. Both ligands crystallize in the *anti* form, as previously observed for Lig^1^H_2_.^39^

Consistent with the solid-state structures, both ^1^H and ^19^F NMR spectra for the separated products suggest the presence of a single diastereomer. Thus, while two signals for the O*H* protons (at approximately 1:1 intensity) were observed in the proton spectrum of the crude mixture, separated products exhibited single peaks (Figure 2). Similarly, ^19^F NMR of the product mixture demonstrated two signals, whereas single peaks are observed for the isolated diastereomers (). ^13^C NMR spectra for both enantiomers contain a partially resolved quartet around 128 ppm (^1^ *J*_C–F_ = 280 Hz), consistent with the presence of a CF_3_ group. Notably, the central phenyl protons for the homochiral diastereomer *rac*-Lig^2^H_2_ appear as two broad peaks (around 6 ppm) in both benzene and dichloromethane at RT. VT NMR in *d*_8_-toluene (Figure 3) demonstrates coalescence of these peaks into a single peak above 40 °C. These two peaks, additional low-intensity peaks for the central phenyl, and a low-intensity peak for the OH protons (∼2.5 ppm) are observed below −20 °C. *Meso*-Lig^2^H_2_ exhibits similar behavior (). Thus, the central phenyl protons appear as a broad peak at RT and as a single sharp peak at elevated temperatures. A more complicated pattern emerges at low (<−20 °C) temperature, consistent with the presence of two species in a ∼2:1 ratio. This suggests discrete *syn* and *anti* (likely dominating) isomers at low temperature with equilibration above room temperature.

**Figure 3:**
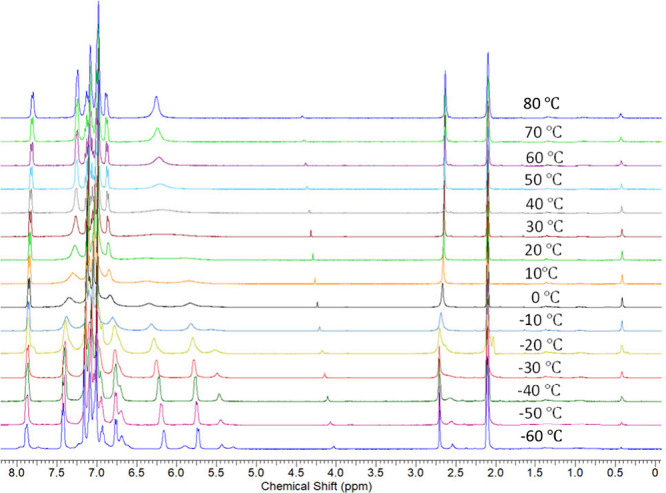
VT NMR spectra of *rac*-Lig^2^H_2_, demonstrating (1) two isomers at low temperature and (2) equilibration above room temperature.

We investigated the coordination chemistry of both ligands with select divalent main-group and transition metals, Mg­(II) and Cr­(II). Previously synthesized *p*-terphenyl-based bis­(alkoxide) ligand Lig^1^H_2_ exhibited chelating behavior (requiring *syn* conformation of the alkoxides) with Cr­(II), Mn­(II), and Fe­(II).^39,43,44^ The related tetra-*tert*-butyl ligand Lig^3^H_2_ failed to form complexes, likely due to excessive sterics and the resulting inability to adopt a *syn* conformation.^45^ Treatment of both isomers of Lig^2^H_2_ with *n*-butyl-*sec*-butylmagnesium led cleanly to formation of mononuclear complexes Mg­(*rac*-Lig_2_)­L_2_ (**1**) and Mg­(*meso*-Lig_2_)­L_2_ (**2**), in which the ligand is *syn* and chelating (Figure 4). While the complexes are obtained initially as THF adducts (L = THF), subsequent recrystallization from ether can result in the substitution of THF ligands by diethyl ether. Both complexes were characterized by ^1^H, ^13^C, ^19^F NMR and IR spectroscopy (see ) and X-ray crystallography. While **1** crystallized as an expected bis­(THF) complex, complex **2** exhibited ether ligation at one of the positions. The structure revealed distorted tetrahedral geometry, with wider O1–Mg–O2 (interalkoxide) angles of 129.9(1)° for **1** and 129.80(4) for **2** and a narrower O3–Mg–O4 (interether) angle of 95.1(1)° for both **1** and **2**. These metrics are typical for the related Mg­(OR)_2_(THF)_2_ complexes with bulky alkoxide ligands.^37,38^

**Figure 4:**
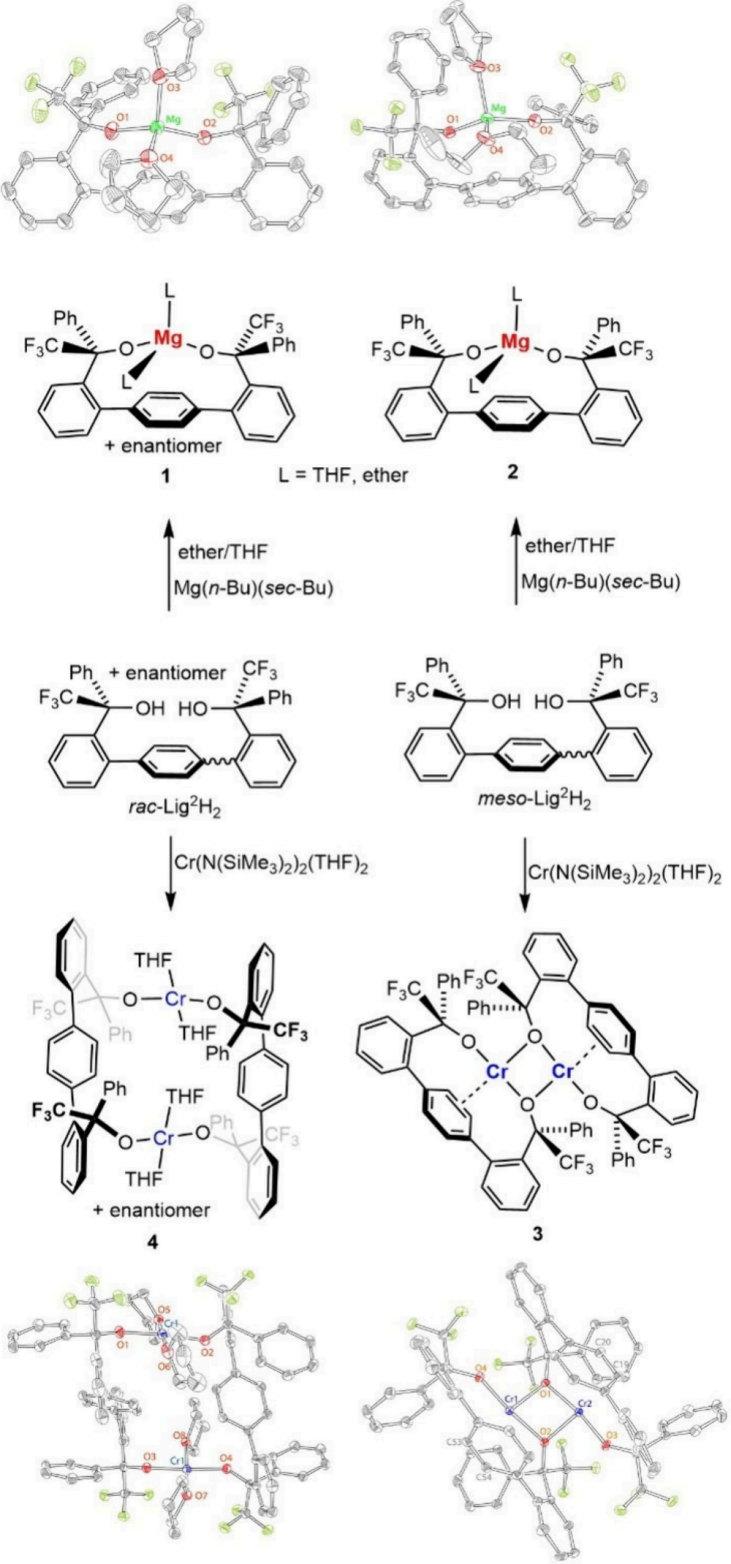
Reactions of *rac*/*meso*-Lig^2^H_2_ to produce the corresponding mononuclear Mg­(II) and dinuclear Cr­(II) products. X-ray structures of **1**–**4** are drawn using ORTEP, with 50% probability ellipsoids.

In contrast to magnesium, reaction of the chromium precursor Cr­(N­(SiMe_3_)_2_)_2_(THF)_2_ ^46^ with *rac*/*meso*-Lig^2^H_2_ led to formation of different products, confirmed by X-ray crystallography (Figure 4). The reaction of *meso*-Lig^2^H_2_ with Cr­(N­(SiMe_3_)_2_)_2_(THF)_2_ forms Cr_2_(*meso*-Lig_2_)_2_ dimer (**3**), in which each Lig_2_ coordinates to one chromium center in a chelating fashion, while bridging through one alkoxide oxygen to another chromium. While a similar structural motif [Cr_2_(OR)_4_][Bibr ref43] was obtained for Lig1 and bulky monodentate alkoxide/siloxide ligands,^47–49^ the present structure exhibits square-planar geometry at Cr­(II) (see below) that involves a Cr-arene interaction (based on a relatively short Cr-arene distance of 2.5 Å for **3**; the same distance was 3.0 Å for [Cr_2_(Lig^1^)_2_]).^43^ In contrast to the *meso* ligand, the homochiral ligand led to formation of the bimetallic complex [Cr_2_(*rac*-Lig_2_)_2_(THF)_4_] (**4**), in which each ligand is bridging two chromium centers, in the κ^1^/κ^1^ mode. Both ligands in the same dimer have the same chirality (i.e., SS in Figure 4), resulting in the approximate *D*_2_ symmetry of each dimer molecule. As **4** crystallizes in the *P*2_1_/c space group, the *D*_2_-symmetric enantiomer of the opposite chirality is also found in the unit cell. The chromium­(II) d^4^ centers again exhibit a square-planar geometry, in which the alkoxide donors are trans to each other (173.7(1)/176.6(1)°). Solution magnetic measurements for **3** and **4** (see ) revealed μ_eff_ values of 3.8(4) and 4.3(4) μB, respectively. Lower than expected values for the high-spin di-Cr­(II) complexes can result from antiferromagnetic coupling or accessible low-spin states.

It is noteworthy that while the *C*_2_ and the *C*_s_ symmetric diastereomers of Lig_2_ produce different Cr­(II) structures, both feature square-planar Cr­(II) centers. This is in contrast to the previously reported Cr­(II) complexes in bulky bis­(alkoxide) ligand environments (whether monodentate [OC^*t*^Bu_2_R] (R = H, ^*t*^Bu, Ph, 3,5-Ph_2_C_6_H_3_), closely related [OSi^*t*^Bu_3_], or bidentate Lig_1_), that exhibited trigonal planar or distorted seesaw geometry at Cr­(II).^43,47–50^ It is likely that this difference results from the presence of the trifluoromethyl substituents. Doerrer and co-workers have previously demonstrated that electron-withdrawing fluorinated substituents (in perfluoro-*tert*-butoxide [OC­(CF_3_)_3_]^−^, perfluoropinacolate [{OC­(CF_3_)_2_}_2_]^2–^, and various fluorinated aryloxides) reduce the π-donating ability of the alkoxide ligands.^51–60^ The reduction in π-donicity affects the position of these ligands on the spectrochemical series, making them stronger-field. Our results demonstrate that even a single CF_3_ group is sufficient to significantly modify the electronic character of alkoxide ligation. Due to the increase in their ligand field character, these alkoxides prefer square-planar geometry generally indicative of stronger-field ligands.

To better understand the different coordination chemistry, calculations were performed at the B3LYP-D4/​def2-TZVP/​SMD­(THF)//​BP86/​def2-SVP level of theory (see for full details).^61–76^ Hypothetical monomers Cr­(*rac*/*meso*-Lig_2_) were found to have high-spin Cr­(II) centers and prefer coordination of two THF molecules (), similar to previous complexes.^43,50^ Reaction of these putative monomers to dimerize and form nonet **3**_***rac/meso***_ or **4**_***rac/meso***_ featuring two high-spin Cr­(II) centers was exergonic in each case (Table 1). Thermodynamic preference for the observed crystallographic structure is seen for each ligand by ∼10 kcal/mol over the putative dimer (**3**_***rac***_ and **4**_***meso***_). To better understand this preference, we computed the energy of the ligands preorganizing to their conformation in the dimer structures (ΔE_preorg_), and the interaction energies of the Cr­(II) ions with the preorganized ligands (ΔE_int_) for the overall reaction energy (ΔE_rxn_) as shown in Table 2. For dimers **3**, ΔE_preorg_ is nearly identical at ∼+110 kcal/mol but there is a notable preference in ΔE_int_ for **3**_***meso***_, likely due to weaker Cr–arene interactions (Figure 5) evidenced by longer Cr–C distances of 0.1–0.2 Å for **3**_***rac***_. For dimers **4**, however, ΔE_int_ is nearly identical ∼ −610 kcal/mol, consistent with similar Cr–O bond lengths (), and the preference for **4**_***rac***_ is in ΔE_preorg_. This could be due to (i) organizing the bis­(alkoxides), (ii) organizing the THFs, or (iii) bringing all six ligands together. shows that the difference arises from ΔE_preorg_ for the bis­(alkoxide) ligands. The conformation of *rac*-Lig2 in **4**_***rac***_ is nearly identical to that in the free diol ligand (Figure 2), while *meso*-Lig2 in **4**_***meso***_ requires one of the CF_3_ groups to rotate into a position above the middle phenyl of the bridging terphenyl group, disrupting the favorable π-stacking observed in the free ligand (Figure 6).

**Figure 5:**
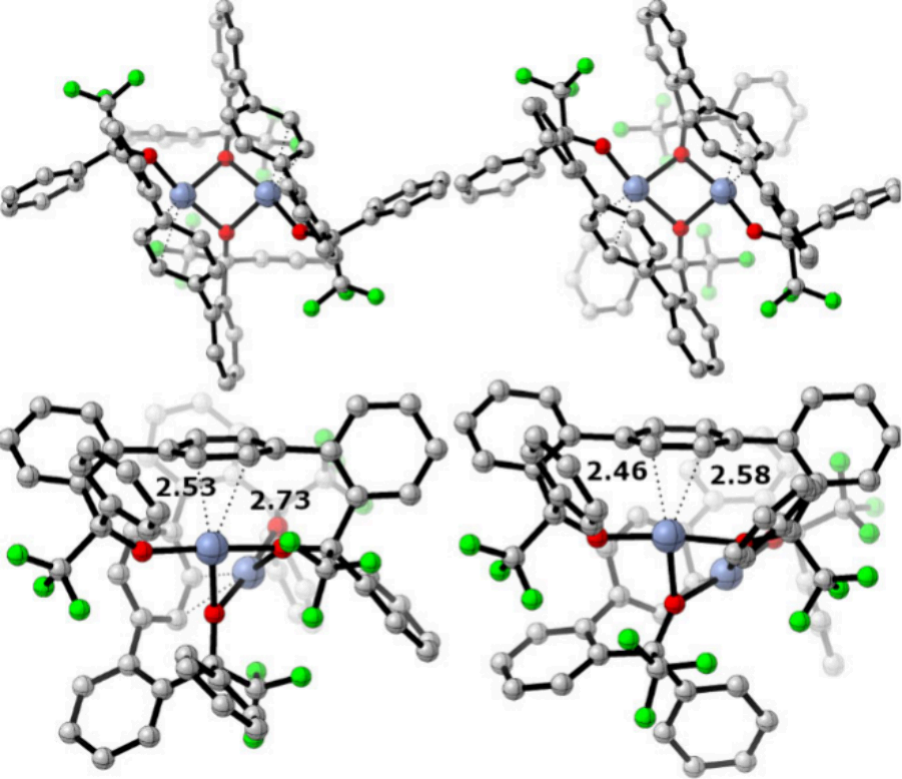
Optimized structures of **3**_***rac***_ (top left) and **3**_***meso***_ (top right), with side-on views of the Cr–arene interaction, with distances (Å) below each structure.^77^ Hydrogens omitted for clarity.

**Figure 6:**
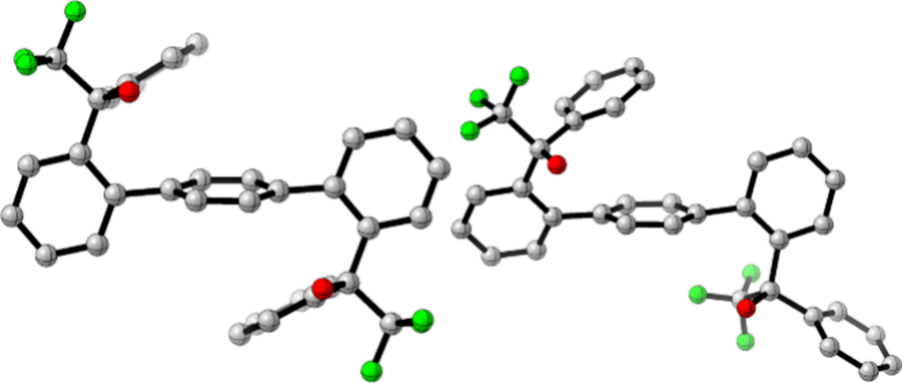
Ligand conformations of *rac*-Lig2 (left) and *meso*-Lig2 (right) from **4**_***rac***_ and **4**_***meso***_, respectively.^77^ Full dimer structures are in the Supporting Information ().

**Table 1:** Dimerization Thermodynamics in kcal/mol

Reaction	ΔG
2 Cr(*rac*-Lig2)(THF)_2_ → **3**_***rac***_ + 4 THF	–16.59
2 Cr(*meso*-Lig2)(THF)_2_ → **3**_***meso***_ + 4 THF	–19.18
2 Cr(*rac*-Lig2)(THF)_2_ → **4**_***rac***_	–27.28
2 Cr(*meso*-Lig2)(THF)_2_ → **4**_***meso***_	–7.65

**Table 2:** Preorganization and Interaction Energies (kcal/mol) to Make These Dimers

Reaction	ΔE_preorg_	ΔE_int_	ΔE_rxn_
2 Cr^2+^ + 2 (*rac*-Lig^2^)^2–^ → **3**_***rac***_	+108.30	–584.36	–476.07
2 Cr^2+^ + 2 (*meso*-Lig^2^)^2–^ → **3**_***meso***_	+111.66	–598.19	–486.53
2 Cr^2+^ + 2 (*rac*-Lig^2^)^2–^ + 4 THF → **4**_***rac***_	+72.25	–611.01	–528.48
2 Cr^2+^ + 2 (*meso*-Lig^2^)^2–^ + 4 THF → **4**_***meso***_	+84.36	–608.90	–515.02

In summary, we describe the synthesis and separation of new *C*_2_ and *C*_s_ symmetric bulky bis­(alkoxide) ligands and their coordination chemistry with Mg­(II) and Cr­(II). For Mg­(II), the expected mononuclear *C*_2_- and *C*_s_-symmetric complexes were obtained. In contrast, for Cr­(II) the *C*_s_-symmetric ligand exhibited chelating behavior similar to Lig,^1,43^ whereas the *C*_2_-symmetric ligand bridged two metals in κ^1^-fashion. Both stereoisomers, however, exhibited square-planar geometry at Cr­(II), in contrast to previously reported Cr­(II) complexes with bulky alkoxides. We assign this difference to the electron-withdrawing CF_3_ substituent that turns down the π-donicity of the alkoxides and therefore increases their ligand field. In future studies, we will target other main-group and transition metal complexes of both *rac*- and *meso*-Lig^2^H_2_ and investigate their reactivity.

## Supplementary Material





## Notes

Synthetic procedures, NMR, IR, and mass spectra (PDF)
Structures for all optimized geometries (XYZ)
