# Supplementary material for: The Effect of a Single Trifluoromethyl Substituent on the Reactivity of Chelating C 2 and C s‑Symmetric Bis(alkoxide) Ligands on a Terphenyl Platform
Source: Inorg Chem. 2026 Jan 15;65(4):2110–6. doi: 10.1021/acs.inorgchem.5c05470 (PMC12869483; doi:10.1021/acs.inorgchem.5c05470)
Supplement: Supplementary file 1 [file ic5c05470_si_001.pdf]

Supporting Information

The Effect of a Single Trifluoromethyl Substituent on the Reactivity of Chelating C_2 and C_s-symmetric Bis(alkoxide) Ligands on a Terphenyl Platform

Ruwandhi Jayasundara,[$a] Lakshani W. Kulathungage,[$a] Benjamin J. Baillie,[a] Cassandra L. Ward,[b] Richard L. Lord,*[c] Stanislav Groysman*[a]

E-mails: lordri@gvsu.edu, groysman@wayne.edu

**Table of contents**	**Page**
1. General experimental details	S3
2. Synthetic procedures	S4
3. X-ray crystallographic details and structure of $Cr(N(SiMe_3)_2)_2(THF)_2$	S7
4. NMR spectra	S11
5. HRMS spectra	S30
6. IR spectra	S32
7. Evans method formula and procedure	S36
8. Computational details	S37
9. References	S42

1. General experimental details

Air-sensitive reactions were carried out in a nitrogen-filled glovebox. 1,4-Bis(2-bromophenyl)benzene was synthesized using previously reported procedures.[1] 2-Bromophenylboronic acid, 1,4-diiodobenzene, K_2CO_3, and 2,2,2-trifluoroacetophenone were purchased from Sigma-Aldrich and used as received. Tert-butyllithium (1.7 M in hexanes) was purchased from Sigma-Aldrich, stored and handled in the nitrogen-filled glovebox (see hazard notion below). C_6D_6 and CD_2Cl_2 were purchased from Cambridge Isotope Laboratories and stored over 3 Å molecular sieves. HPLC grade non-deuterated solvents were purchased from Sigma-Aldrich and purified using an MBraun solvent purification system. The ligand precursors were characterized by ^{1}H, ^{13}C NMR, and ^{19}F NMR spectroscopy, high-resolution mass spectrometry, and X-ray crystallography. Metal complexes were generally characterized by ^{1}H, ^{13}C and ^{19}F NMR (if diamagnetic) spectroscopy, solution magnetic moment measurements (if paramagnetic), IR spectroscopy, X-ray crystallography, and elemental analysis. NMR spectra of the ligands and metal complexes were recorded at the Lumigen Instrument Centre on an Agilent 400 MHz Spectrometer and Agilent DD2-600 MHz Spectrometer in C_6D_6 or CD_2Cl_2 at room temperature. Chemical shifts and coupling constants (J) were reported in parts per million and Hertz respectively. Thermo Fisher Scientific LTQ Orbittrap XL mass spectrometer at the Lumigen Instrument Center was used for high resolution mass spectra. Elemental analyses were carried out at Midwest Microlab.

2. Synthetic procedures

Synthesis and characterization of *rac*-Lig^{2}H$_2$ and *meso*-Lig^{2}H$_2$

To a solution of 1,4-bis(2-bromophenyl)benzene (0.670 g, 1.7 mmol) cooled to -35 ºC in THF, a cold solution of t-BuLi (1.7 M in pentane, 4.1 ml, 6.9 mmol) was added dropwise in the drybox while maintaining the temperature. Following the addition, the reaction was allowed to warm to room temperature, stirred for 2 h, and then transferred into a round bottom flask containing a cold solution of 2,2,2-trifluoroacetophenone (0.5 ml, 3.5 mmol) in diethyl ether. The reaction was stirred for 24 h, followed by aqueous workup to form the protonated ligand. The organic layer was separated, dried with Na_2SO_4, and filtered, followed by solvent evaporation. The crude product was first purified through column chromatography (8% ethyl acetate in hexanes) to obtain a mixture of diastereomers as a white solid in 62% yield (0.620 g). The diastereomers were then separated by column chromatography (5% diethyl ether in hexanes) to produce the *rac*-Lig^{2}H$_2$ as a white solid in 36% yield (0.360 g, 0.622 mmol) and the *meso*-Lig^{2}H$_2$ as a white solid in 26% yield (0.260 g, 0.449 mmol). The recrystallization of both isomers in diethyl ether yielded colorless crystals and the structures were confirmed by X-ray crystallography.

Caution! Tert-butyllithium is extremely pyrophoric. It must be handled under dry conditions using proper needle and syringe techniques. In this work, tert-butyllithium was used and stored in the N_2-filled glovebox.

***rac*-Lig^{2}H$_2$**. ^{1}H NMR (C_6D_6, 600 MHz) δ 7.86 (d, J = 6 Hz, 2H, Ph), 7.32 (m, 4H, Ph), 7.09 (m, 3H, Ph), 7.02 (m, 7H, Ph), 6.88 (m, 2H, Ph), 6.38 (m, 2H, Ph), 5.93 (m, 2H, Ph), 2.72 (s, 2H, OH) ppm. ^{1}H NMR (CD_2Cl_2, 600 MHz) δ 7.76 (d, J = 6 Hz, 2H, Ph), 7.45 (m, 4H, Ph), 7.24 (m, 10H, Ph), 7.11 (d, J = 6 Hz, 2H, Ph), 6.67 (m, 2H, Ph), 6.12 (m, 2H, Ph), 2.97 (s, 2H, OH) ppm. ^{13}C NMR (CD_2Cl_2, 150 MHz) δ 142.45, 140.30, 140.49, 137.51, 133.94, 129.03, 128.92, 128.80, 128.52, 128.41, 127.96, 127.83, 127.66, 125.7 (q, $^1J_{C\text{-}F}$ = 280 Hz, *C*F$_3$), 81.46 (*C*(O)CF$_3$) ppm. ^{19}F NMR (CD_2Cl_2, 600 MHz) δ -73.46 ppm. HRMS (m/z): Calcd, $[C_{30}H_{20}O_8{\cdot}NH_4]^+$ 596.2024, found 596.2013. Anal. Calcd for $C_{34}H_{24}F_6O_2$: C, 70.59; H, 4.18. Found: C, 70.05; H, 4.28.

***meso*-Lig^{2}H$_2$**. ^{1}H NMR (C_6D_6, 600 MHz) δ 7.86 (d, J = 6 Hz, 2H, Ph), 7.21 (m, 4H, Ph), 7.10 (m, 4H, Ph), 6.95 (m, 8H, Ph), 6.21 (m, 4H, Ph), 2.74 (s, 2H, OH) ppm. ^{1}H NMR (CD_2Cl_2, 400 MHz) δ 7.78 (d, J = 8 Hz, 2H, Ph), 7.44 (m, 4H, Ph), 7.24 (m, 10H, Ph), 7.13 (d, J = 8 Hz, 2H, Ph), 6.50 (m, 4H, Ph), 3.00 (s, 2H, OH) ppm. ^{13}C NMR (CD_2Cl_2, 100 MHz) δ 142.38, 140.39, 137.83, 133.74, 133.20, 129.00, 128.76, 128.45, 128.13, 127.69, 127.60, 127.57, 125.6 (q, $^1J_{C\text{-}F}$ = 283 Hz,

CF_3), 81.29 (C(O)CF_3) ppm. ^{19}F NMR (CD_2Cl_2, 600 MHz) δ -73.28 ppm. HRMS (m/z): Calcd, $[C_{34}H_{24}F_6O_2·NH_4]^+$ 596.2024; found, 596.1996. Anal. Calcd for $C_{34}H_{24}F_6O_2$: C, 70.59; H, 4.18. Found: C, 69.94; H, 4.23.

Mg(*rac*-Lig^2)(THF)$_2$ (1). A solution of ligand precursor *rac*-Lig^{2}H$_2$ (31 mg, 0.054 mmol, 1.0 equiv.) was cooled to -35 °C in diethyl ether and added dropwise to a cold solution of Mg(n-butyl)(sec-butyl) (7.75 mg, 0.056 mmol, 1.0 equiv.) in hexane. Then, 0.5 mL of THF was added to the reaction mixture. The reaction mixture was stirred for 4 hours, upon which the volatiles were removed *in vacuo*. The resulting solid can be recrystallized from the minimal amount of diethyl ether or the mixture of CH_2Cl_2/ether at −35 °C. The product Mg(*rac*-Lig^2)(THF)$_2$ (**1**) was obtained as colorless X-ray quality crystals in 51% yield (20 mg, 0.027 mmol). ^{1}H NMR (C_6D_6, 400 MHz) δ 7.71 (d, J = 7 Hz, 2H, Ph), 7.62 (d, J = 7 Hz, 4H, Ph), 7.43 (m, 2H, Ph), 7.20 (t, J = 8 Hz, 4H, Ph), 7.14 (m, 4H, Ph), 7.07 (t, J =7 Hz, 2H, Ph), 7.01(d, J = 8 Hz, 2H, Ph), and 6.75 (d, J = 7 Hz, 2H, Ph). ^{13}C NMR (C_6D_6, 400 MHz) δ 149.10, 144.18, 143.44, 142.18, 131.12, 129.32, 127.88, 127.64, 127.40, 127.20, 126.25, 126.16, 125.84, 83.96 (C(O)CF_3), 68.78, 68.15, 24.59, and 14.76 ppm. The CF_3 signal (that should appear as quartet) is likely obscured by very strong C_6D_6 resonances. ^{19}F NMR (C_6D_6, 400 MHz) δ -73.20 ppm. IR (cm^{-1}) 2970 (w), 2153 (w), 1651 (w), 1150 (s), 1049 (m), 910 (w), 756 (s), 702 (s). The structure of the complex was also confirmed by X-ray crystallography. Anal. Calcd for $C_{42}H_{38}F_6MgO_4·CH_2Cl_2$: C, 62.23; H, 4.86. Found: C, 60.91; H, 5.30. The compound shows lower than expected percentage in C, which is likely due to the air-sensitive and hygroscopic nature of **1**, that leads to the substitution of THF ligands by H_2O.

Mg(*meso*-Lig^2)(THF)$_2$ (**2**). A solution of ligand precursor *meso*-Lig^{2}H$_2$ (35 mg, 0.060 mmol, 1.0 equiv.) was cooled to -35 °C. in diethyl ether and added dropwise to a cold solution of Mg(n-butyl)(sec-butyl) (8.7 mg, 0.063 mmol, 1.0 equiv.) in hexane. Then, 0.5 mL of THF was added to the reaction mixture. The reaction mixture was stirred for 4 hours, upon which the volatiles were removed *in vacuo*. The resulting solid can be recrystallized from the minimal amount of ether or CH_2Cl_2/ether at −35 °C. The product Mg(*meso*-Lig^2)(THF)$_2$ (**2**) was obtained as colorless X-ray quality crystals in 52% yield (23 mg, 0.031 mmol). ^{1}H NMR (C_6D_6, 400 MHz) δ 7.87 (d, J = 8 Hz, 2H, Ph), 7.72 (d, J = 8 Hz, 4H, Ph), 7.43 (dd, J = 7 Hz, 2H, Ph), 7.30-7.25 (m, 6H, Ph), 7.18-7.13 (m, 7H, Ph), and 6.55 (s, 2H, Ph) ppm. ^{13}C NMR (C_6D_6, 400 MHz) δ 149.09, 143.81, 142.75,

142.61,131.33, 129.66, 128.66, 127.88, 127.66, 127.40, 127.34, 126.75, 126.37, 126.12, 125.48, 83.57 (*C*(O)CF_3), 68.67, 65.41, 24.65, and 15. ppm. The *C*F_3 signal (that should appear as quartet) is likely obscured by very strong C_6D_6 resonances. ^{19}F NMR (C_6D_6, 400 MHz) δ -73.29 ppm. IR (cm^{-1}) 2924 (w), 1443 (w), 1221 (m), 1119 (s), 1033 (m), 930 (m), 758 (m). Anal. Calcd for $C_{42}H_{38}F_6MgO_4{\cdot}CH_2Cl_2$: C, 62.23; H, 4.86. Found: C, 62.98; H, 5.22. The structure of the complex was also confirmed by X-ray crystallography.

Cr_2(*meso*-Lig^2)$_2$ (**3**). A solution of ligand precursor *meso*-Lig^2H_2 (33.4 mg, 0.058 mmol, 1.0 equiv.) was cooled to -35 °C in THF and added dropwise to a cold solution of $Cr(N(SiMe_3)_2)_2(THF)_2$ (30.1 mg, 0.058 mmol, 1.0 equiv.) in THF. The color of the reaction changed from light purple to light green over a course of 4 hours. The volatiles were removed *in vacuo*, and the resulting solid was recrystallized from the minimal amount of CH_2Cl_2/ether at −35 °C. Cr_2(*meso*-Lig^2)$_2$ (**3**) was obtained as very pale blue X-ray quality crystals in 53% yield (20 mg, 0.031 mmol). IR (cm^{-1}) 2927 (w), 1444 (w), 1254 (m), 1134 (s), 1043 (m), 920 (m). μ_{eff} = 3.8 μ_B. Anal. Calcd for $C_{70}H_{50}Cr_2F_{12}O_4{\cdot}2CH_2Cl_2$: C, 58.92; H, 3.39 Found: C, 59.07; H, 3.75. The structure of the complex was confirmed by X-ray crystallography (contains two CH_2Cl_2 molecules in the asymmetric unit).

Cr_2(*rac*-Lig^2)$_2$(THF)$_4$ (**4**). A solution of ligand precursor *rac*-Lig^2H_2 (32.6 mg, 0.056 mmol, 1.0 equiv.) was cooled to -35 °C in THF and added dropwise to a cold solution of $Cr(N(SiMe_3)_2)_2(THF)_2$ (29 mg, 0.056 mmol, 1.0 equiv.) in THF. The color of the reaction changed from light purple to light blue to light green over a course of 4 hours. The volatiles were removed *in vacuo*, and the resulting residue was recrystallized from the minimal amount of CH_2Cl_2/ether at −35 °C, which afforded x-ray quality very pale purple (nearly colorless) crystals of Cr_2(*rac*-Lig^2)$_2$(THF)$_4$ (**4**) in 57% yield (25 mg, 0.032 mmol). IR (cm^{-1}) 2870 (w), 1651 (m), 1543 (m), 1147 (s), 1049 (m), 756 (s), 702 (s). μ_{eff} = 4.3 μ_B Elemental analysis indicated consistently low C percentage, which is likely due to the particularly air-sensitive nature of compound **3** (has 4 coordinated THF molecules vs. THF-lacking dimer **4**). The structure of the complex was confirmed by the X-ray structure determination.

3. X-ray crystallographic details

The structures of ligand precursors *rac*-Lig^2H_2, *meso*-Lig^2H_2, and complexes **1**-**4** were determined by X-ray crystallography. Suitable crystals were collected on a Bruker D8 Venture diffractometer with kappa geometry, an Incoatec IμS micro-focus source X-ray tube (Mo K_α radiation), and a multilayer mirror for monochromatization. The data was processed using APEXsoftware. The structures were solved and refined using SHELXT[2] or olex2.solve and difference Fourier (ΔF) maps, as embedded in SHELXL-2019/3[3] or olex2.refine running under Olex2.[4] The hydrogen atoms were placed in calculated positions using a standard riding model and refined isotropically; all other atoms were refined anisotropically. The experimental crystallographic parameters are reported in Tables S1 and S2.

The structure of $Cr(N(SiMe_3)_2)_2(THF)_2$ was originally published by Hursthouse and coworkers.[7] We synthesized this compound as previously described and recrystallized it from hexane (green solution, purple crystals) in order to verify its composition by the unit cell measurement. However, the unit cell parameters of our structure were somewhat different from the published structure. Therefore, we also solved the structure of this compound; its experimental parameters are also reported in Table S1.

This work made use of the single-crystal XRD that was partially funded by the National Institutes of Health supplement grant #3R01EB027103-02S1.

Table S1. Experimental crystallographic parameters for *rac*-Lig^2H_2, *meso*-Lig^2H_2, and **1**, and **2**.

Complex	*rac*-Lig^2H_2	*meso*-Lig^2H_2	**1**	**2**
Formula	$C_{34}H_{24}F_6O_2$ $\cdot 2C_4H_{10}O$	$0.5\cdot C_{34}H_{24}F_6O_2$	$C_{46}H_{48}F_6MgO_5$	$C_{42}H_{40}F_6MgO_4$ $\cdot 0.5(C_4H_{10}O)$
Fw, g/mol	726.81	289.28	819.15	784.11
Temperature, K	100	100	100	100
Crystal System	triclinic	monoclinic	triclinic	triclinic
Space Group	*P*-1	$P2_1$/c	*P*-1	*P*-1
Color	colorless	colorless	colorless	colorless
Z	2	2	2	2
a, Å	11.5711(7)	5.9481(11)	11.2906(7)	10.8014(11)
b, Å	11.8280(7)	27.064(5)	13.2024(8)	12.5348(15)
c, Å	13.9039(9)	8.5814(15)	14.6360(9)	15.9774(18)
α, deg	87.607(2)	90.00	81.586(2)	98.850(4)
β, deg	88.255(2)	104.422(5)	76.810(2)	108.499(4)
γ, deg	75.439(2)	90.00	84.517(2)	100.475(4)
V, A^3	1839.8(2)	1337.9(4)	2096.9(2)	1964.7(4)
d_{calcd}, g/cm^3	1.47	1.436	1.297	1.325
μ, mm^{-1}	0.103	0.117	0.114	0.118
R_1^a (all data)	0.0742	0.1250	0.0968	0.0720
wR_2^b (all data)	0.1131	0.1944	0.2666	0.1481
R_1^a [(I>2σ)]	0.0400	0.0665	0.0862	0.0503
wR_2^b [(I>2σ)]	0.0944	0.1600	0.2592	0.1344
GOF (F^2)	1.0607	1.0961	1.111	1.041

[a] $R_1 = \sum||F_o - |F_c||/\sum|F_o|$. [b] $wR_2 = (\sum (w(F_o^2 - F_c^2)^2)/\sum(w(F_o^2)^2))^{1/2}$. [c] GOF $= (\sum w(F_o^2 - F_c^2)^2/(n - p))^{1/2}$ where n is the number of data and p is the number of parameters refined.

Table S2. Experimental crystallographic parameters for $Cr(N(SiMe_3)_2)_2(THF)_2$, **3**, and **4**.

Complex	$Cr(N(SiMe_3)_2)_2(THF)_2$	**3**	**4**
Formula	$C_{20}H_{52}CrN_2O_2Si_4$	$C_{70}H_{50}Cr_2F_{12}O_4$ $\cdot 2CH_2Cl_2$	$C_{84}H_{76}Cr_2F_{12}O_8$ $\cdot C_4H_{10}O$
Fw, g/mol	516.986	1426.88	1619.56
Temperature, K	100	100	100
Crystal System	triclinic	triclinic	monoclinic
Space Group	*P*-1	*P*-1	$P2_1$/c
Color	light violet	colorless	colorless
Z	2	2	4
a, Å	10.7645(5)	12.8148(6)	20.2881(9)
b, Å	11.3597(5)	14.4315(6)	15.8286(6)
c, Å	13.0941(6)	17.8674(9)	24.9327(12)
α, deg	69.835(2)	84.278(2)	90.00
β, deg	78.878(2)	84.977(2)	104.553(2)
γ, deg	85.349(2)	64.8570(10)	90.00
V, A^3	1474.61(12)	2972.6(2)	7749.8(6)
d_{calcd}, g/cm^3	1.164	1.594	1.388
μ, mm^{-1}	0.568	0.636	0.368
$R_1{}^a$ (all data)	0.0347	0.1236	0.0807
$wR_2{}^b$ (all data)	0.0736	0.1932	0.1162
$R_1{}^a$ [(I>2σ)]	0.0288	0.0741	0.0468
$wR_2{}^b$ [(I>2σ)]	0.0706	$wR_2 = 0.1674$	0.0991
GOF (F^2)	1.0132	1.095	1.007

[a] $R_1 = \sum||F_o - |F_c||/\sum|F_o|$. [b] $wR_2 = (\sum (w(F_o^2 - F_c^2)^2)/\sum(w(F_o^2)^2))^{1/2}$. [c] GOF $= (\sum w(F_o^2 - F_c^2)^2/(n - p))^{1/2}$ where n is the number of data and p is the number of parameters refined.

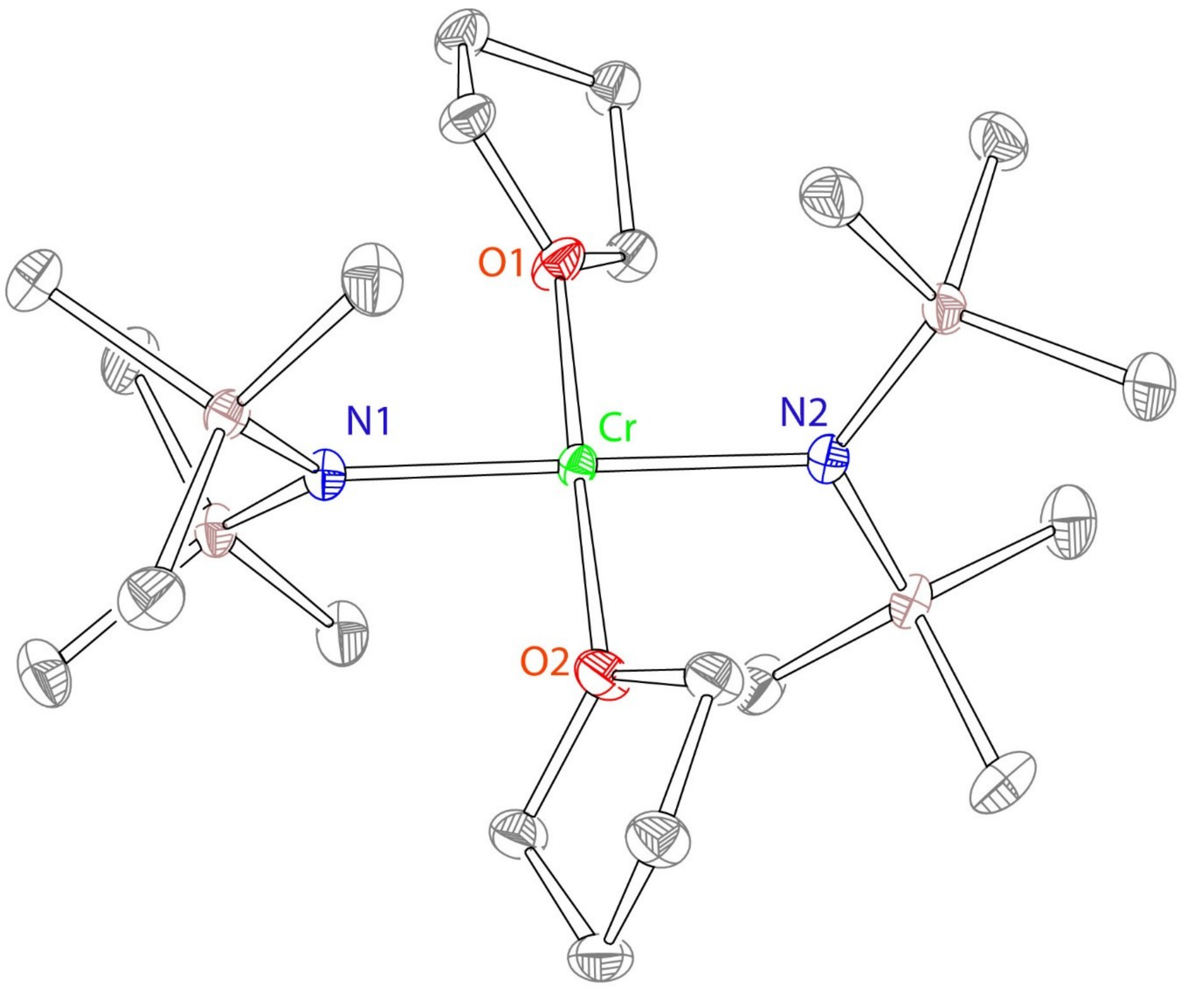

Figure S1. X-ray structure (ORTEP drawing, 50% ellipsoids) of $Cr(N(SiMe_3)_2)_2(THF)_2$. Hydrogen atoms were omitted from clarity.

4. NMR Spectra

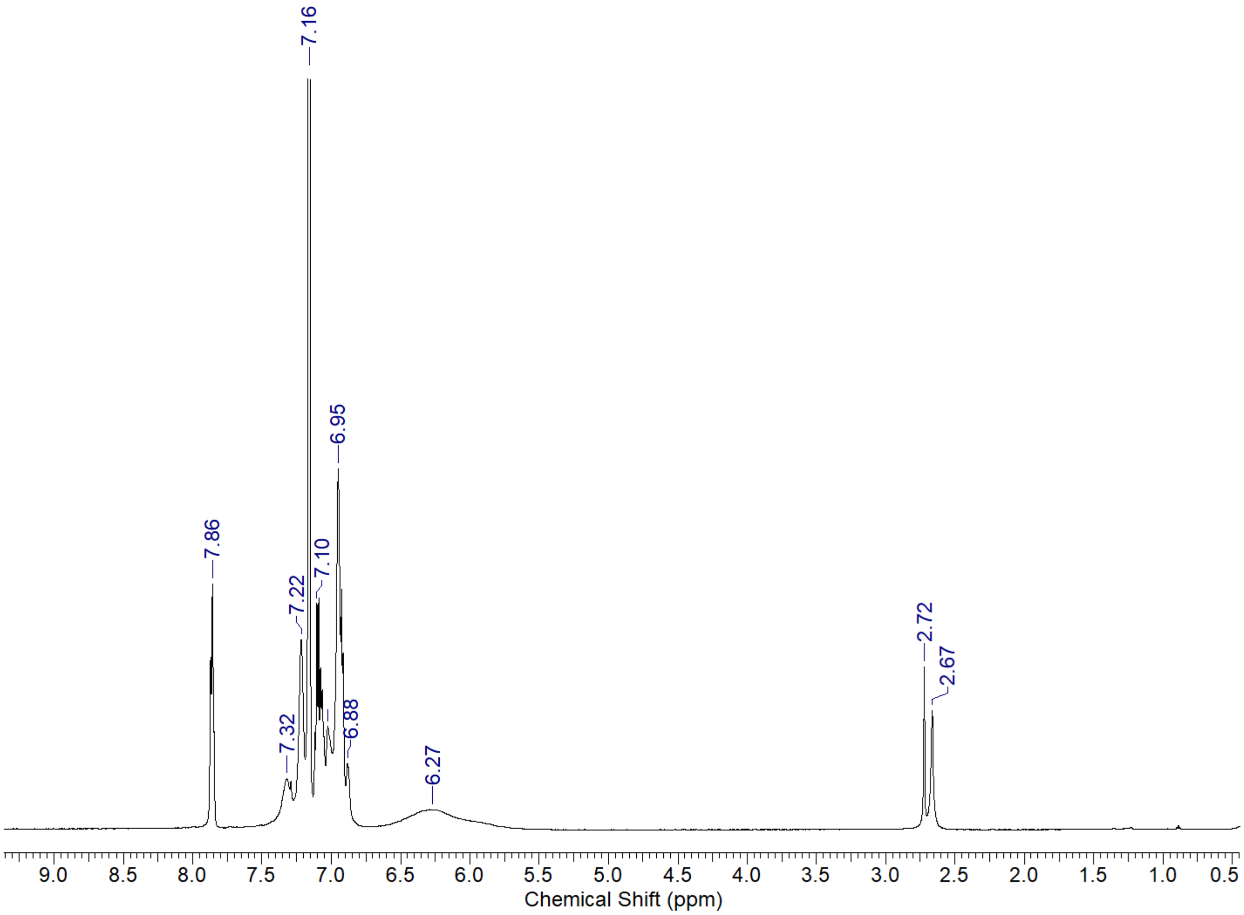

Figure S2. ^{1}H NMR demonstrating the mixture of diastereomers of Lig^{2}H$_2$ (C_6D_6, 600 MHz).

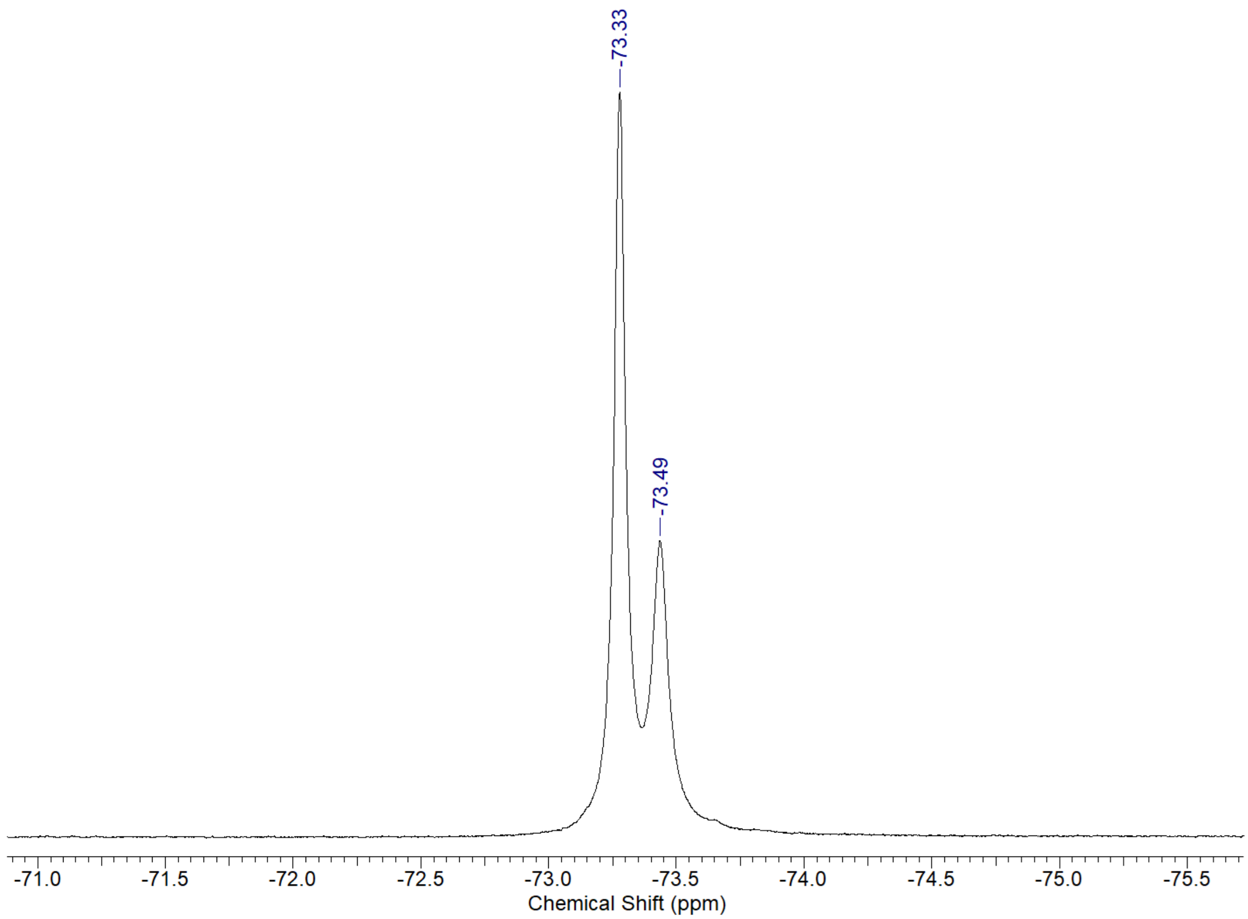

Figure S3. ^{19}F NMR demonstrating the mixture of diasteromers of Lig^{2}H$_2$ (C_6D_6, 600 MHz, 50 °C).

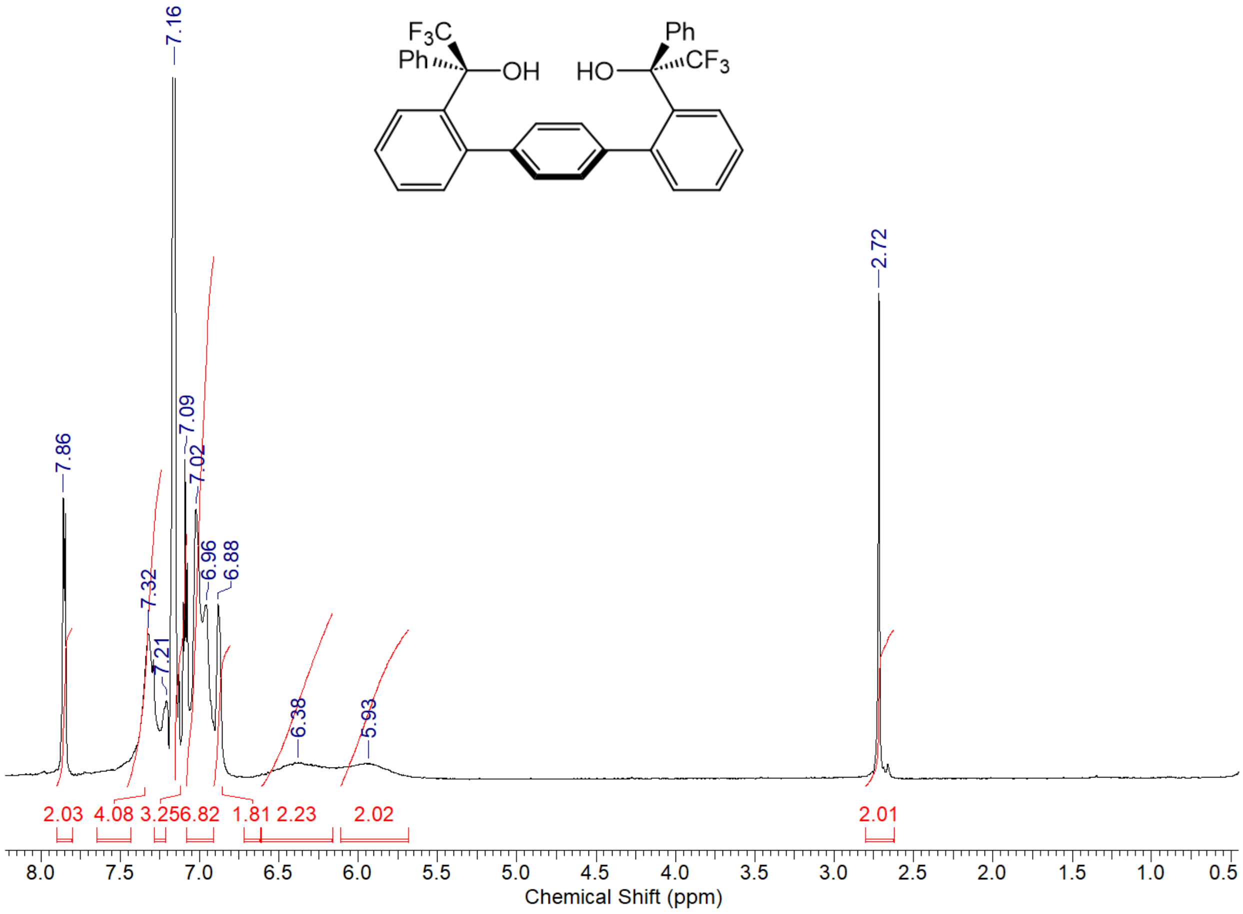

Figure S4. ^{1}H NMR of *rac*-Lig^{2}H$_2$ (C_6D_6, 600 MHz).

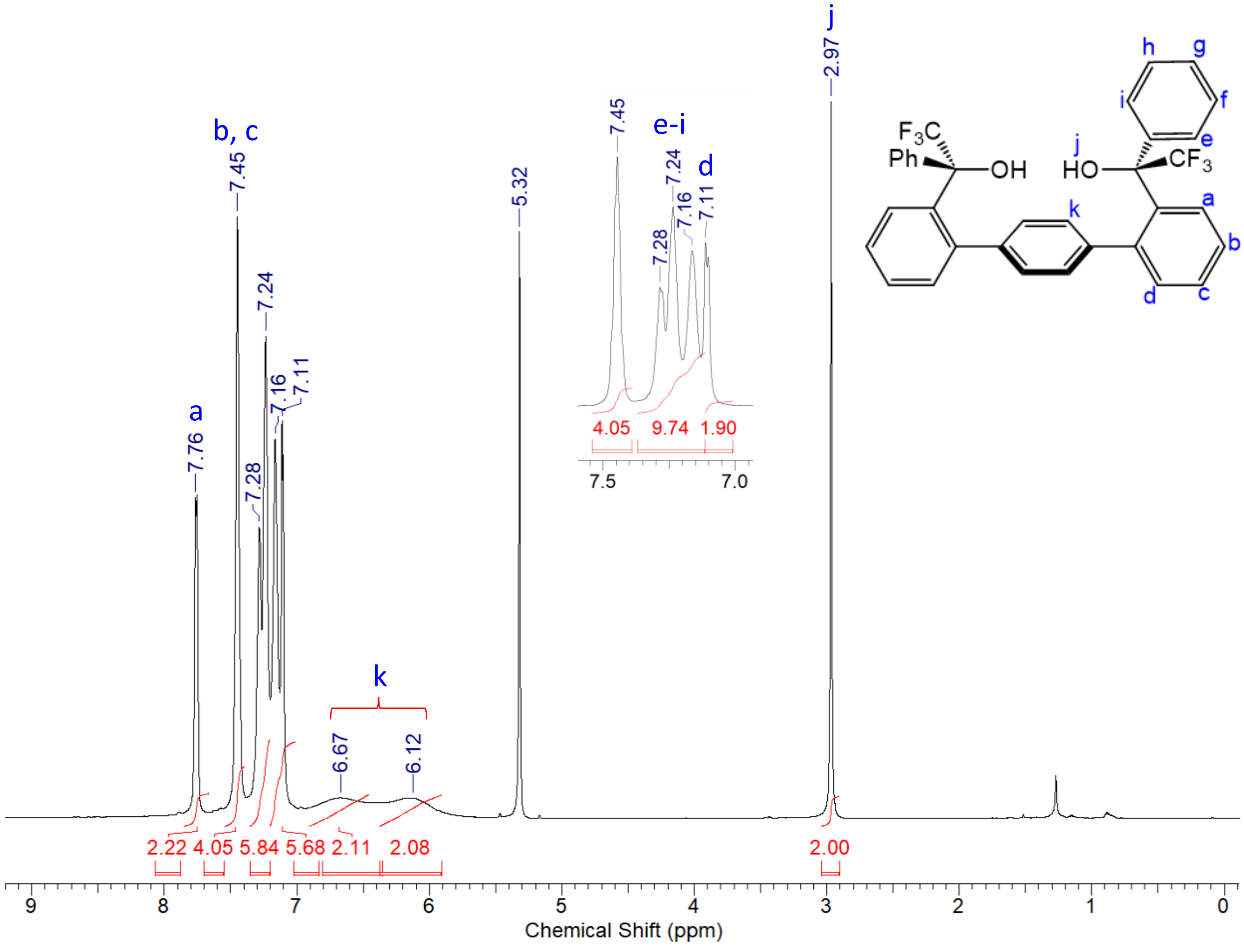

Figure S5. ^{1}H NMR of *rac*-Lig^{2}H$_2$ (CD_2Cl_2, 600 MHz).

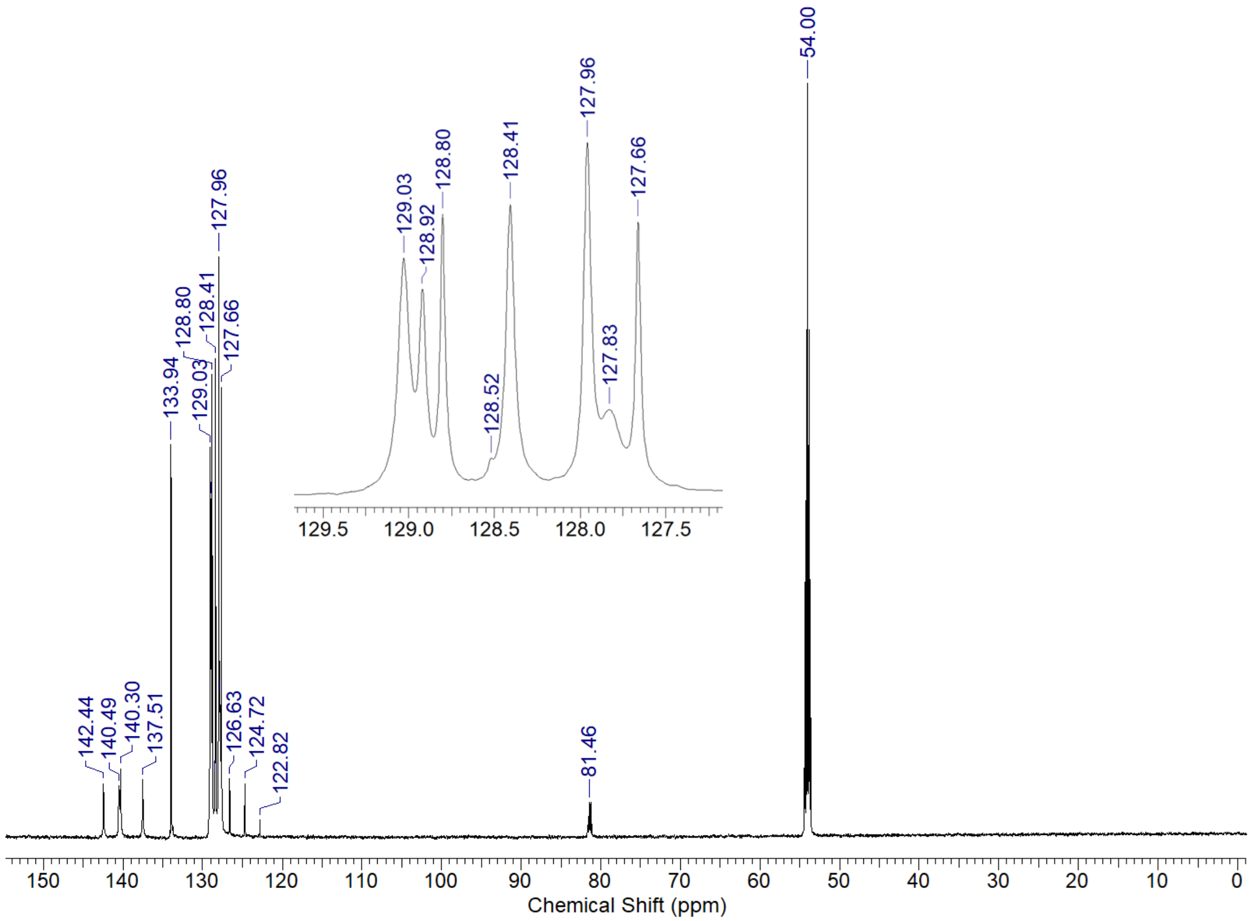

Figure S6. $^{13}C\{^{1}H\}$ NMR of *rac*-Lig^2H_2 (CD_2Cl_2, 150 MHz).

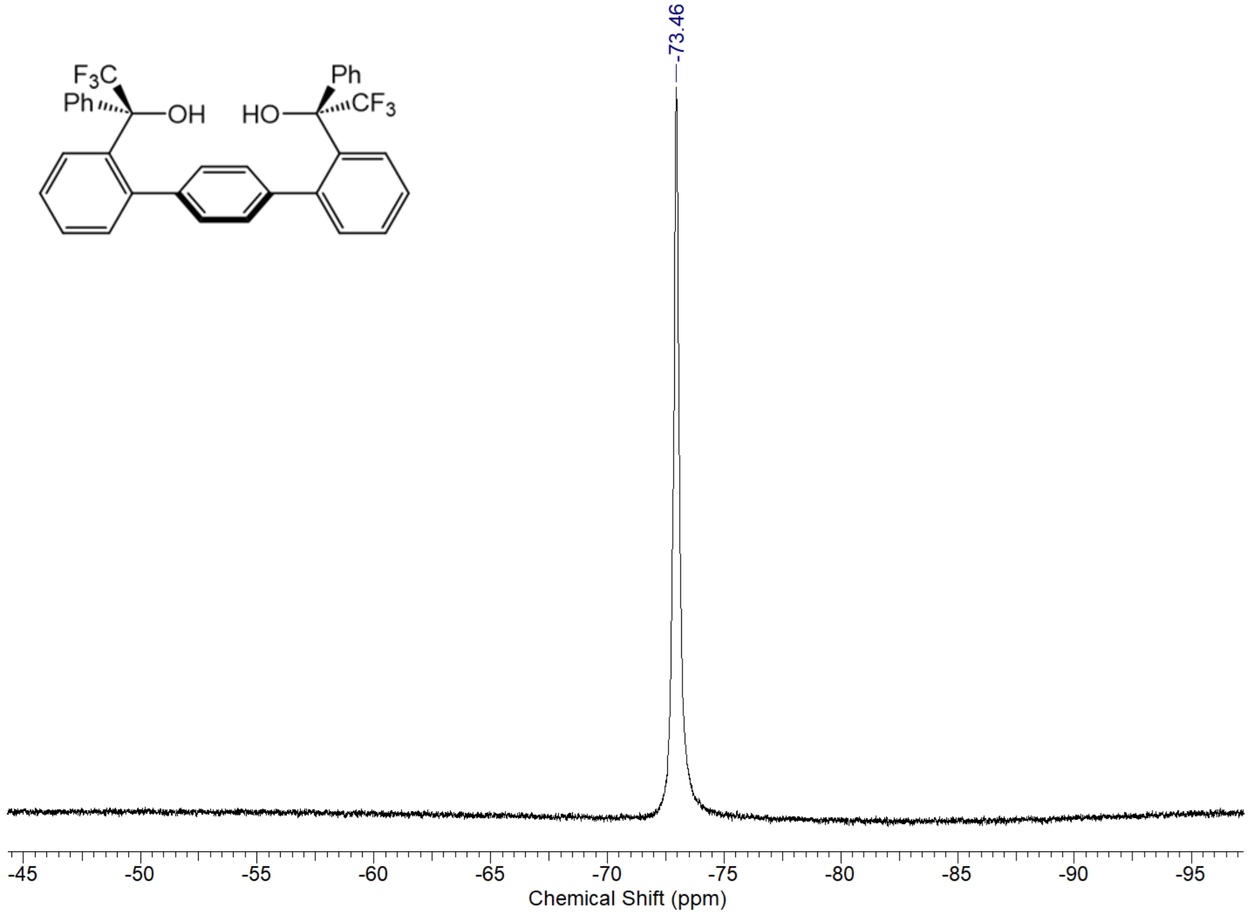

Figure S7. ^{19}F NMR of *rac*-Lig^2H_2 (C_6D_6, 600 MHz).

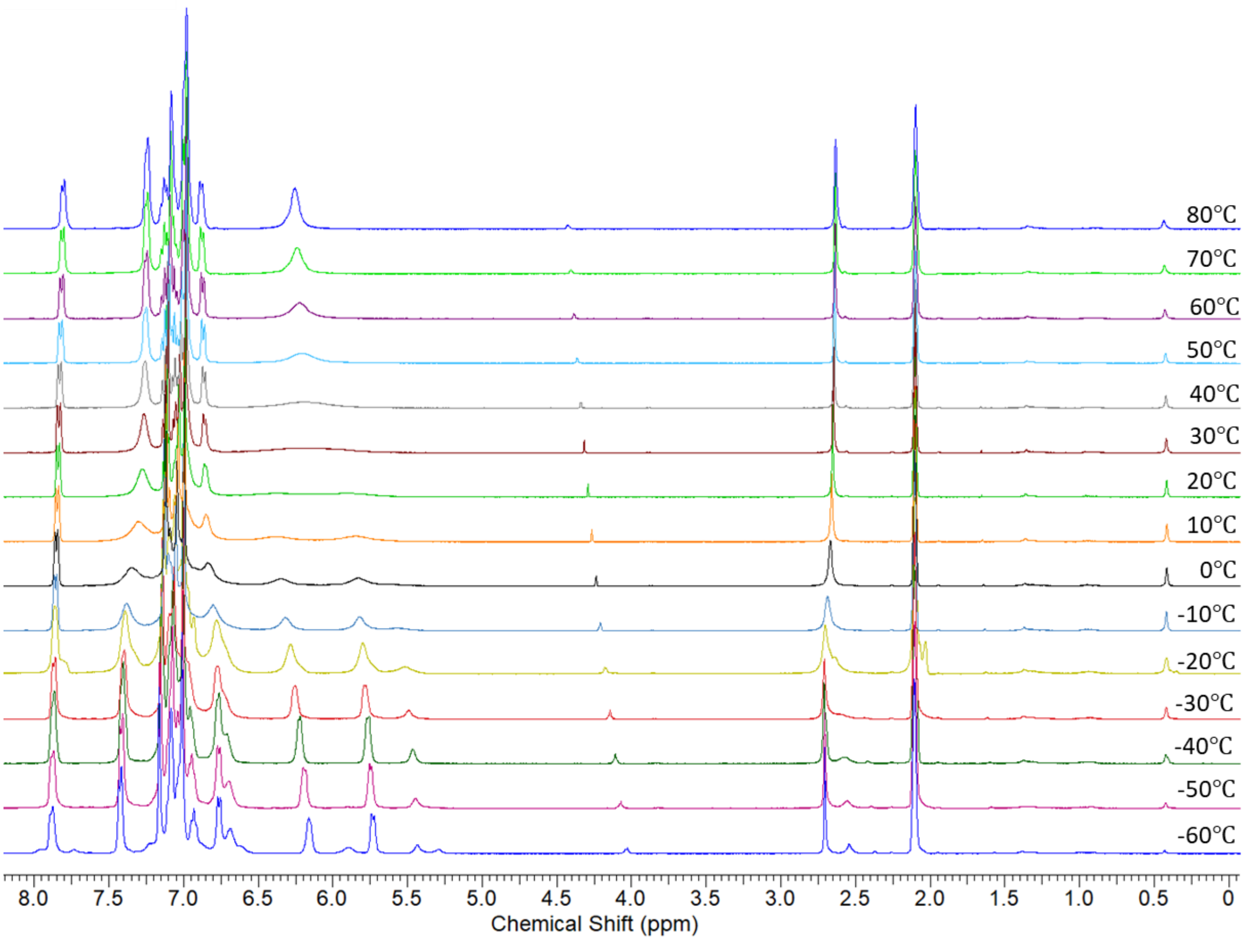

Figure S8. VT ^1H NMR of *rac*-Lig^2H_2 (C_7D_8, 400 MHz).

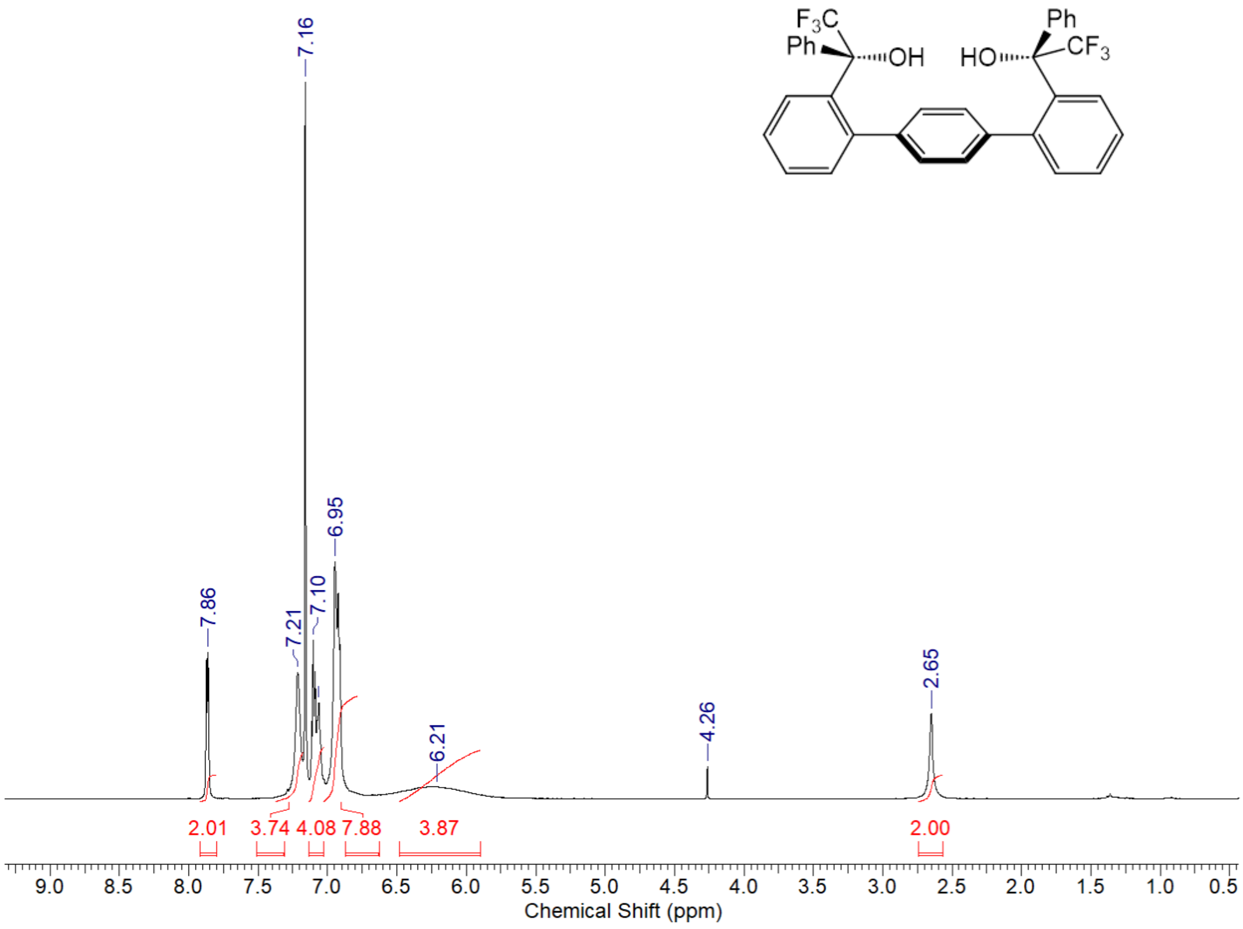

Figure S9. ^{1}H NMR of *meso*-Lig^2H_2 (C_6D_6, 600 MHz).

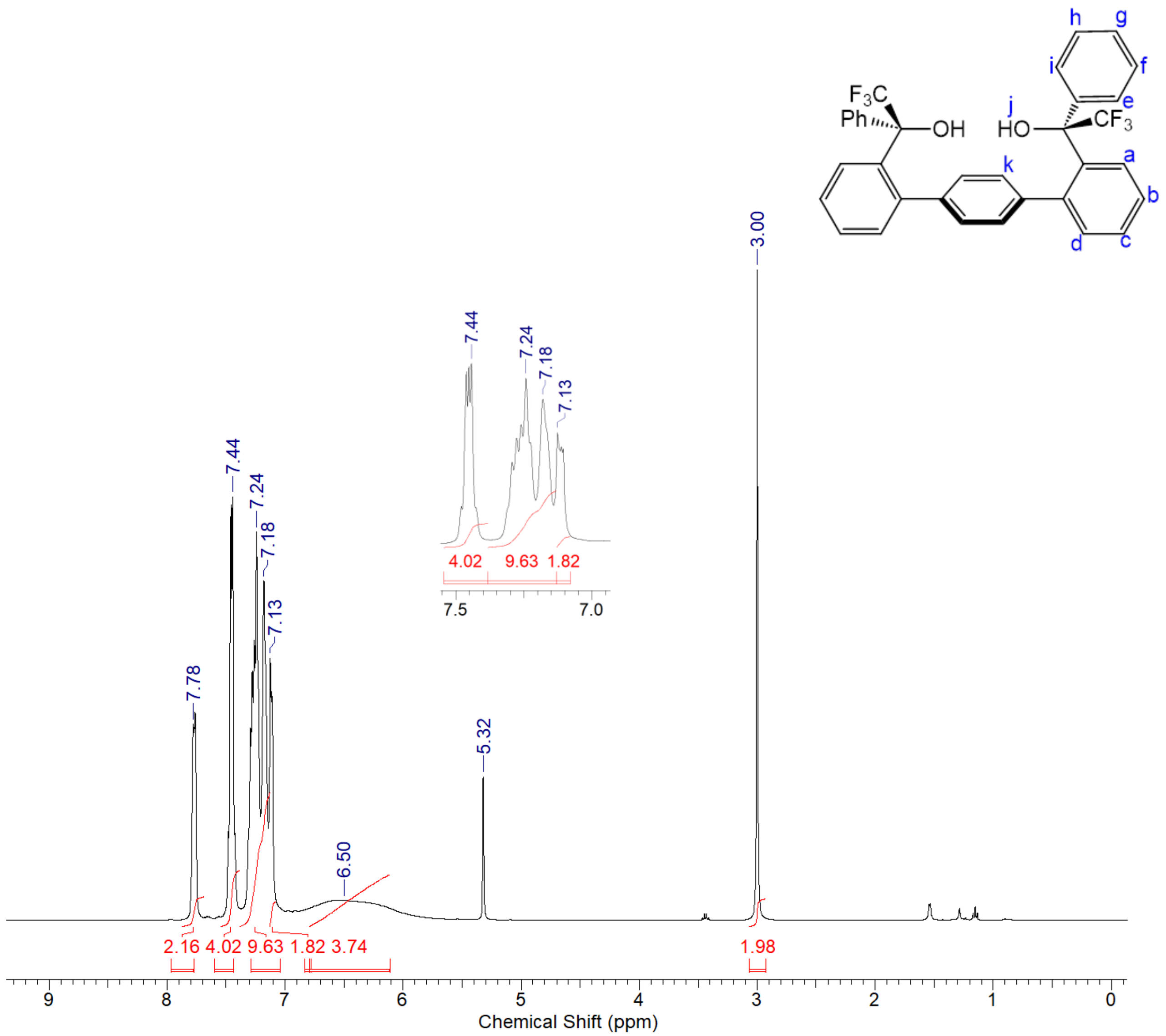

Figure S10. ^{1}H NMR of *meso*-Lig^{2}H$_2$ (CD_2Cl_2, 600 MHz).

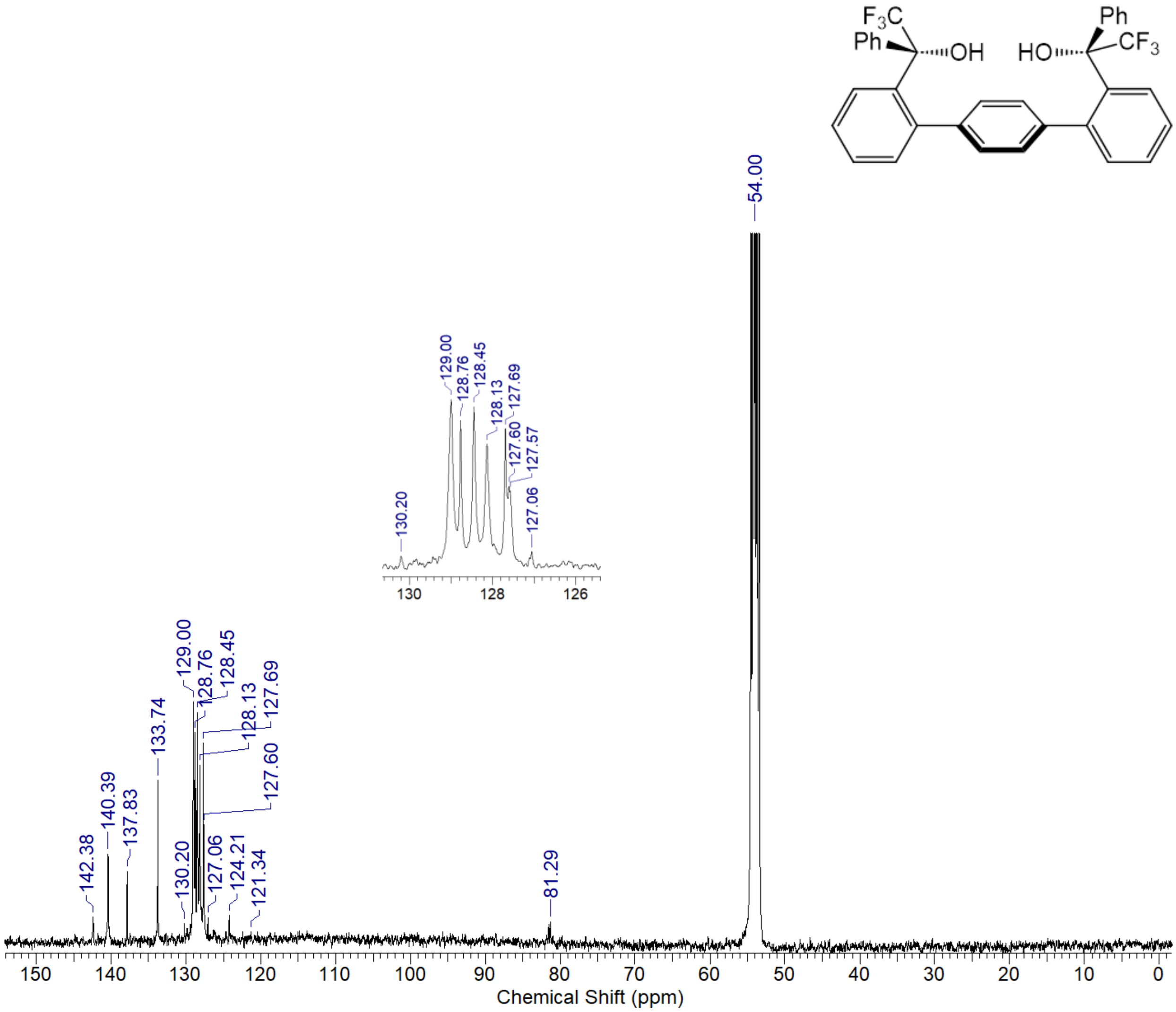

Figure S11. ^{13}C {^{1}H} NMR of *meso*-Lig^2H_2 (CD_2Cl_2, 100 MHz).

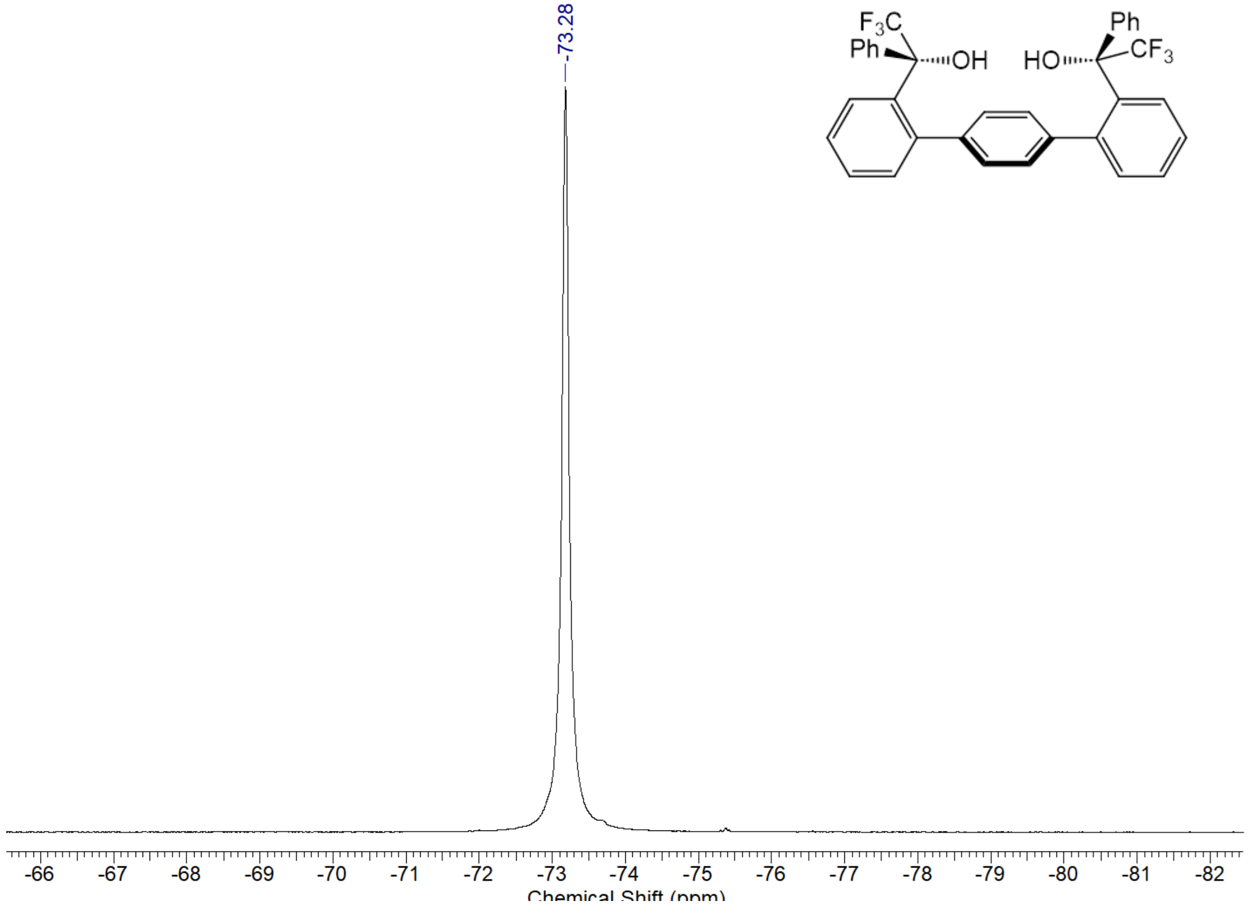

Figure S12. ^{19}F NMR of *meso*-Lig^{2}H$_2$ (C_6D_6, 600 MHz).

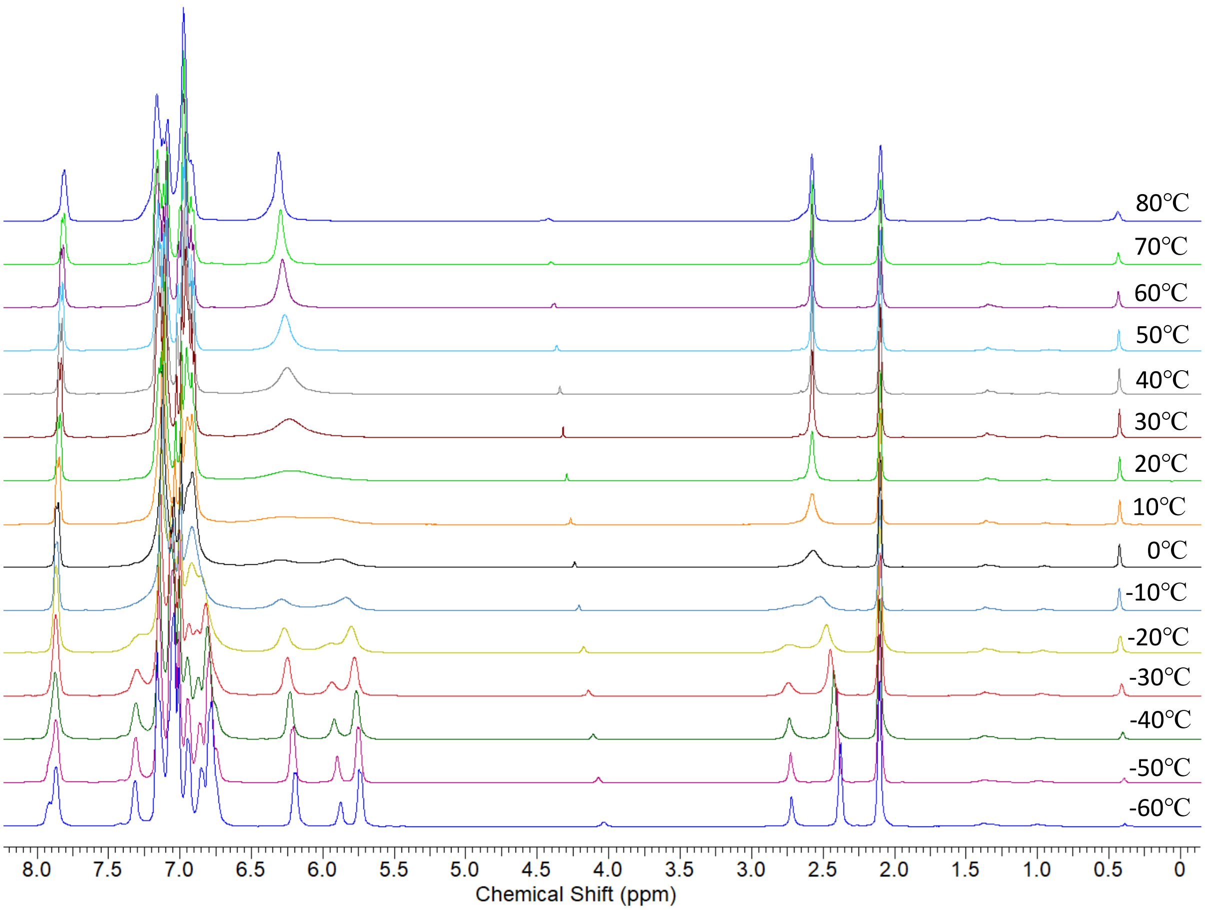

Figure S13. VT ^{1}H NMR of *meso*-Lig^{2}H$_2$ (C_7D_8, 400 MHz).

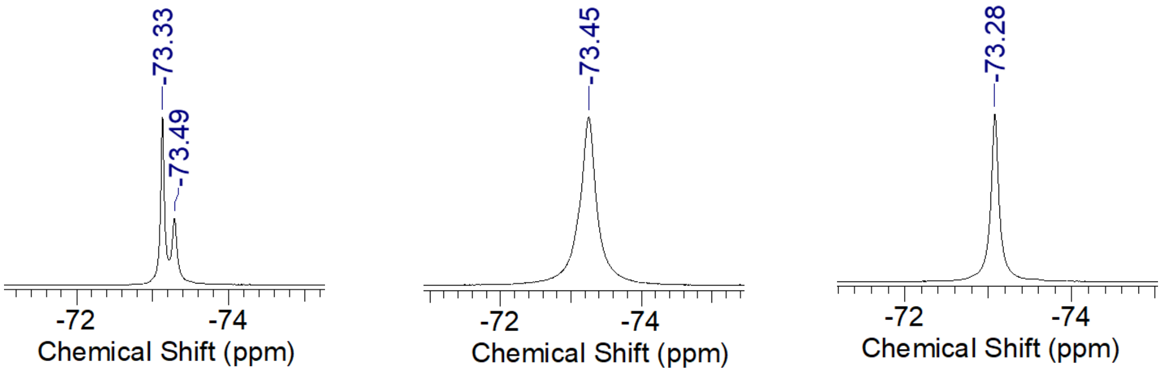

Figure S14. ^{19}F NMR of (a) diastereomers mixture at 50 °C, (b) *rac*-Lig^2H_2 at room temperature, and (c) *meso*-Lig^2H_2 at room temperature (C_6D_6, 600 MHz).

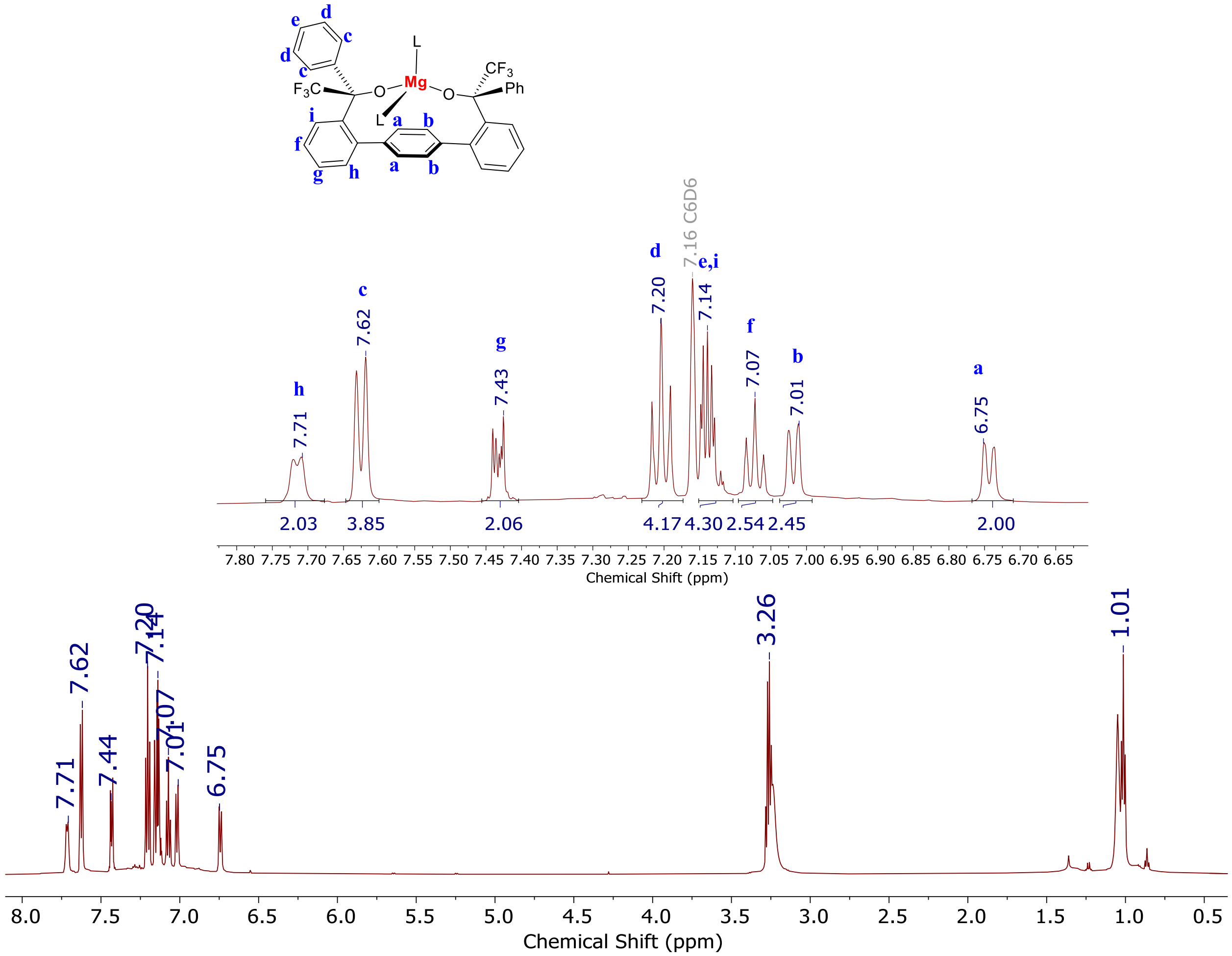

Figure S15. ^{1}H NMR (aromatic region) spectra of Mg(*rac*-Lig^{2})(THF)$_2$ (**1**) at room temperature (C_6D_6, 400 MHz).

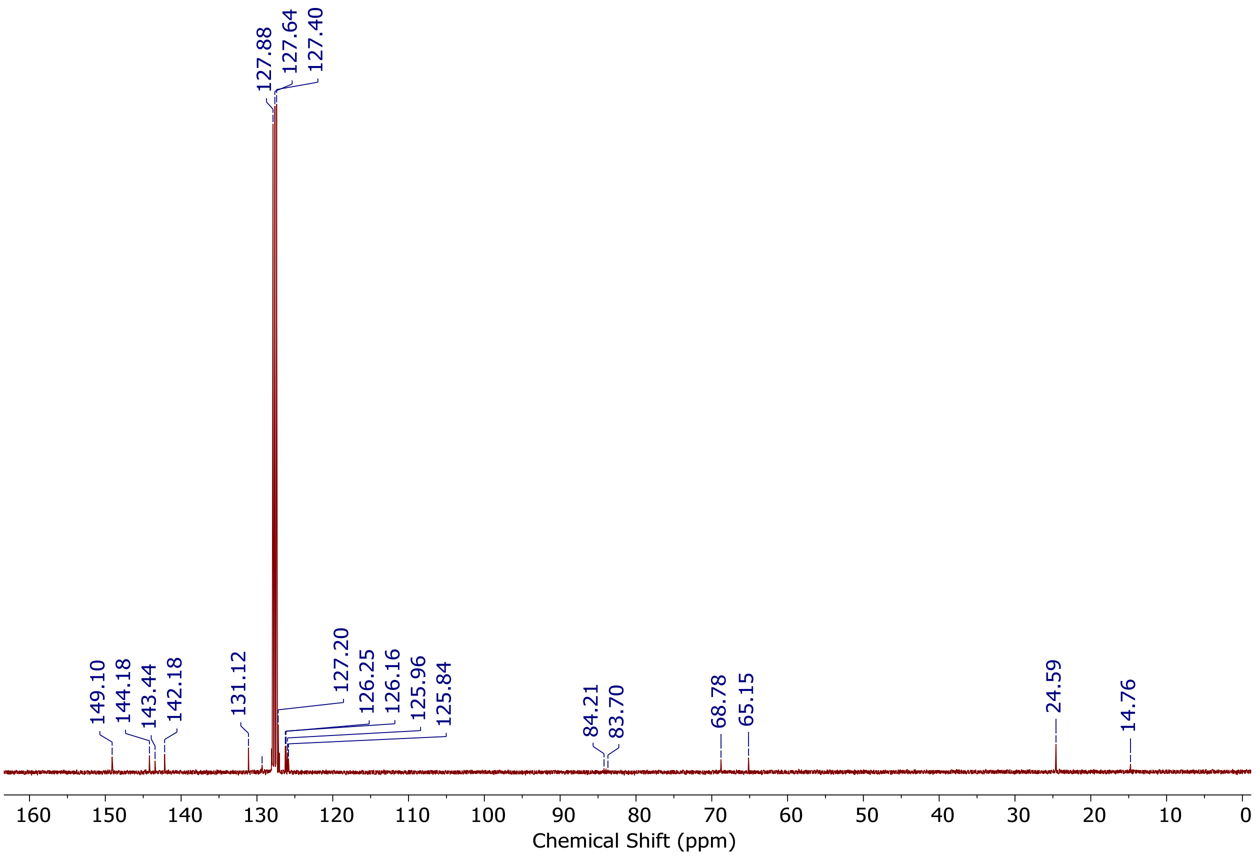

Figure S16. ^{13}C {^{1}H} NMR of Mg(*rac*-Lig^{2})(THF)$_2$ (**1**) (C_6D_6, 400 MHz).

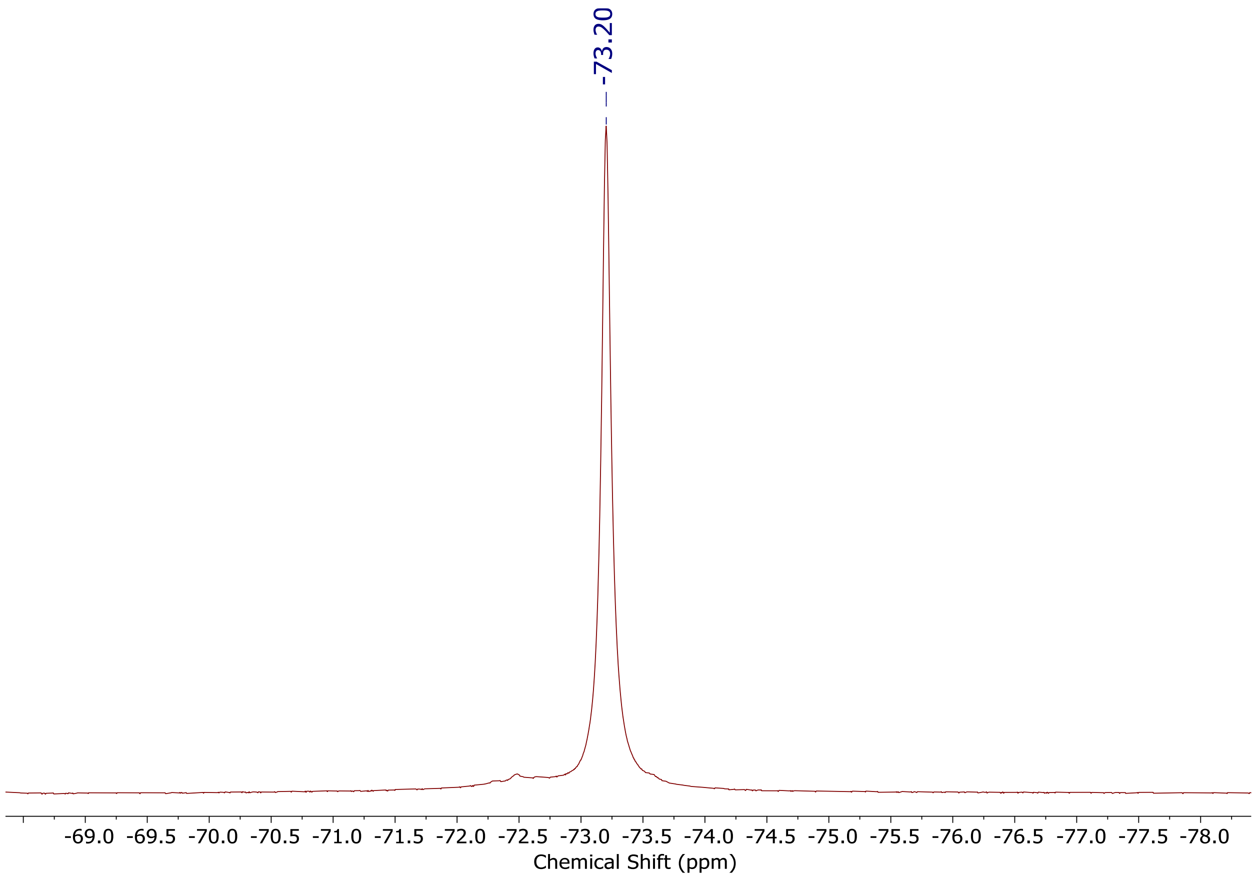

Figure S17. ^{19}F NMR spectra of Mg(*rac*-Lig^2)(THF)$_2$ (**1**) at room temperature (C_6D_6, 400 MHz).

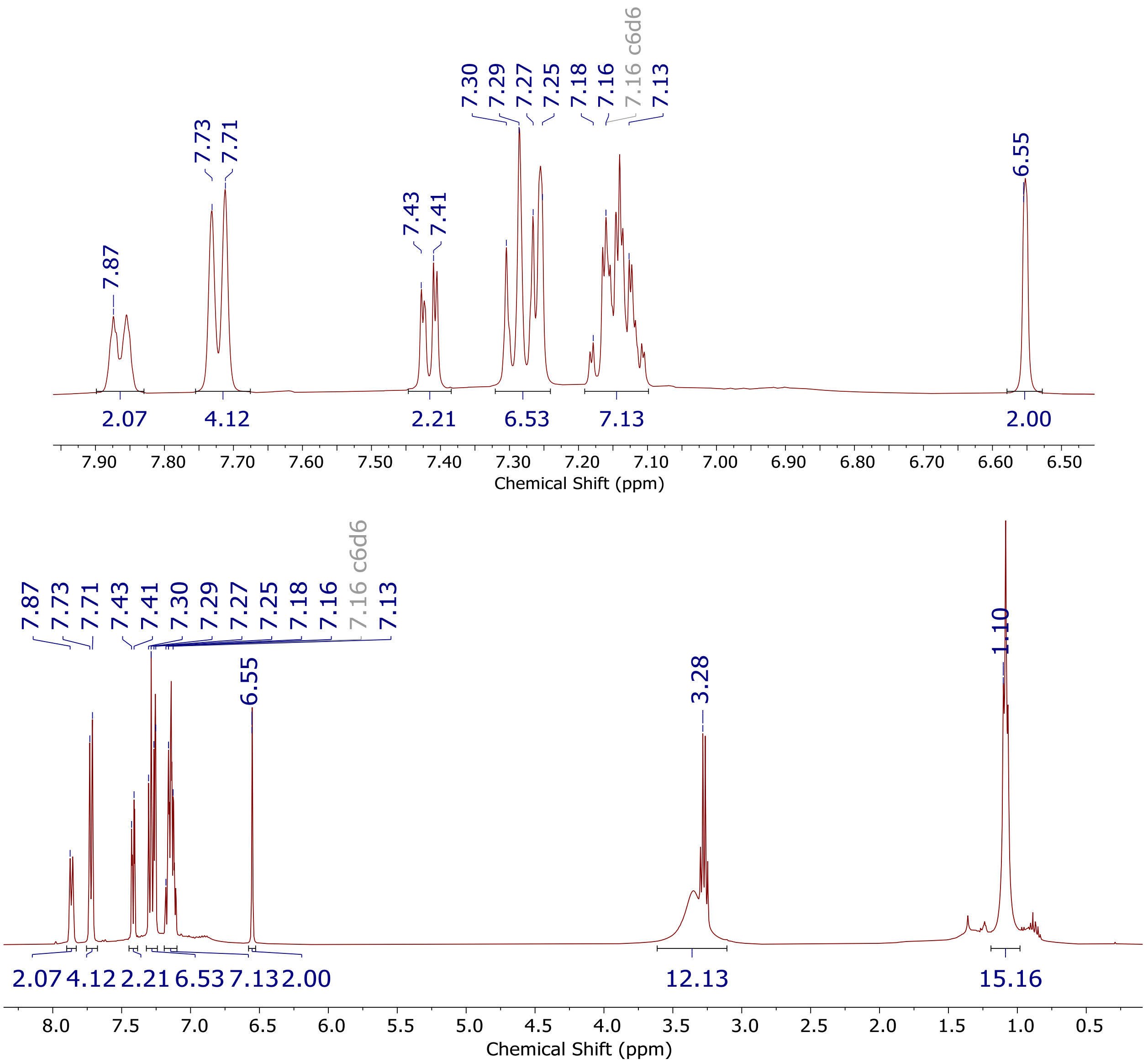

Figure S18. ^{1}H NMR (aromatic region) spectrum of Mg(*rac*-Lig^2)(THF)$_2$ (**1**) at room temperature (C_6D_6, 400 MHz).

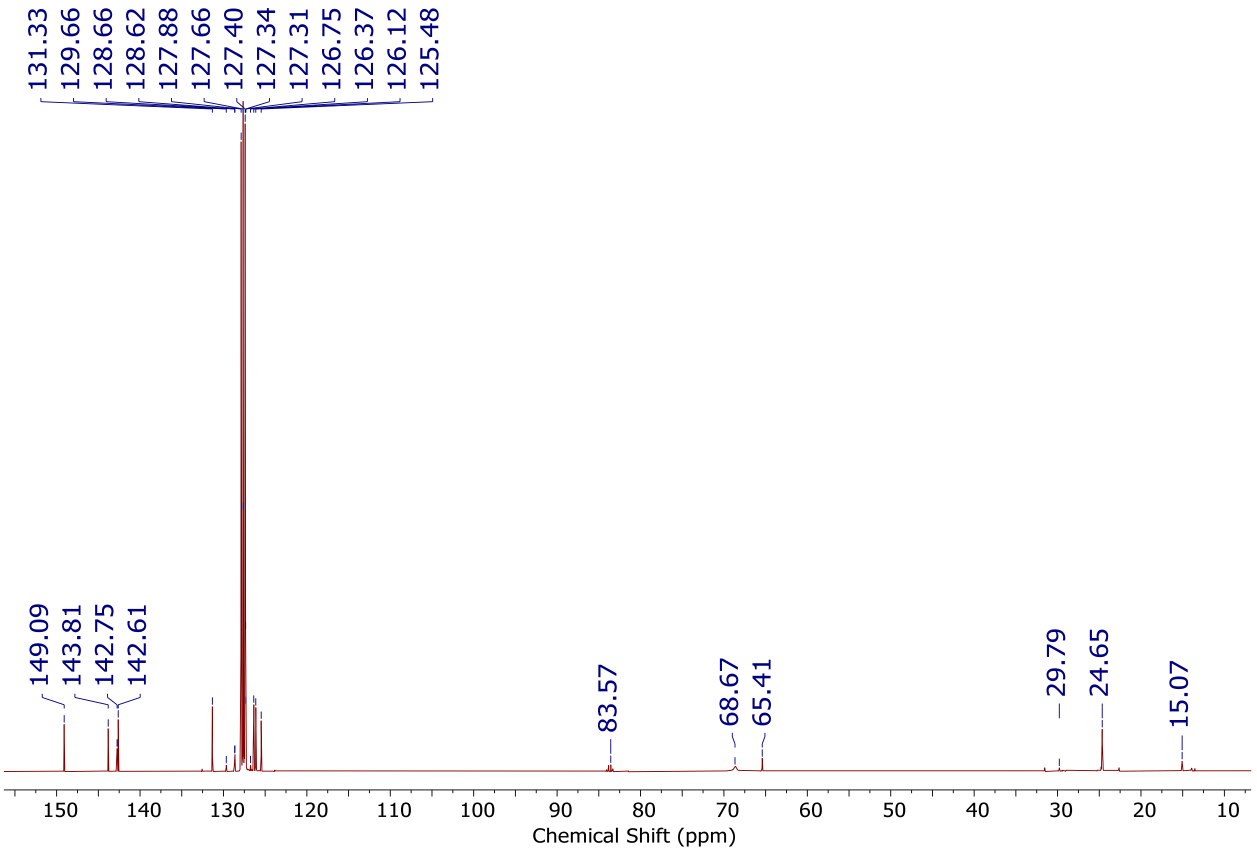

Figure S19. ^{13}C {^{1}H} NMR of Mg(*meso*-Lig^{2})(THF)$_2$ (**2**) (C_6D_6, 400 MHz).

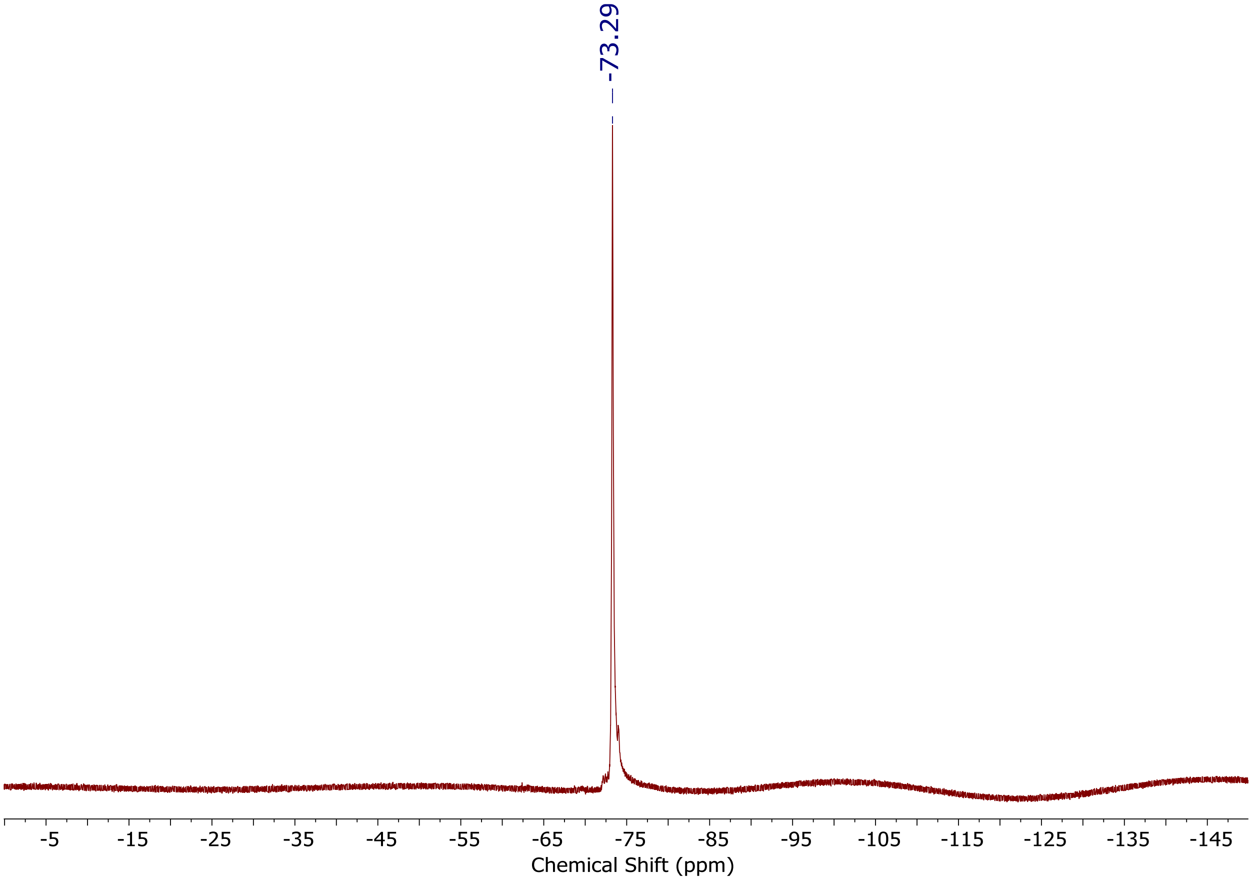

Figure S20. ^{19}F NMR spectra of Mg(*meso*-Lig^{2})(THF)$_2$ (**2**) at room temperature (C_6D_6, 400 MHz).

5. HRMS spectra

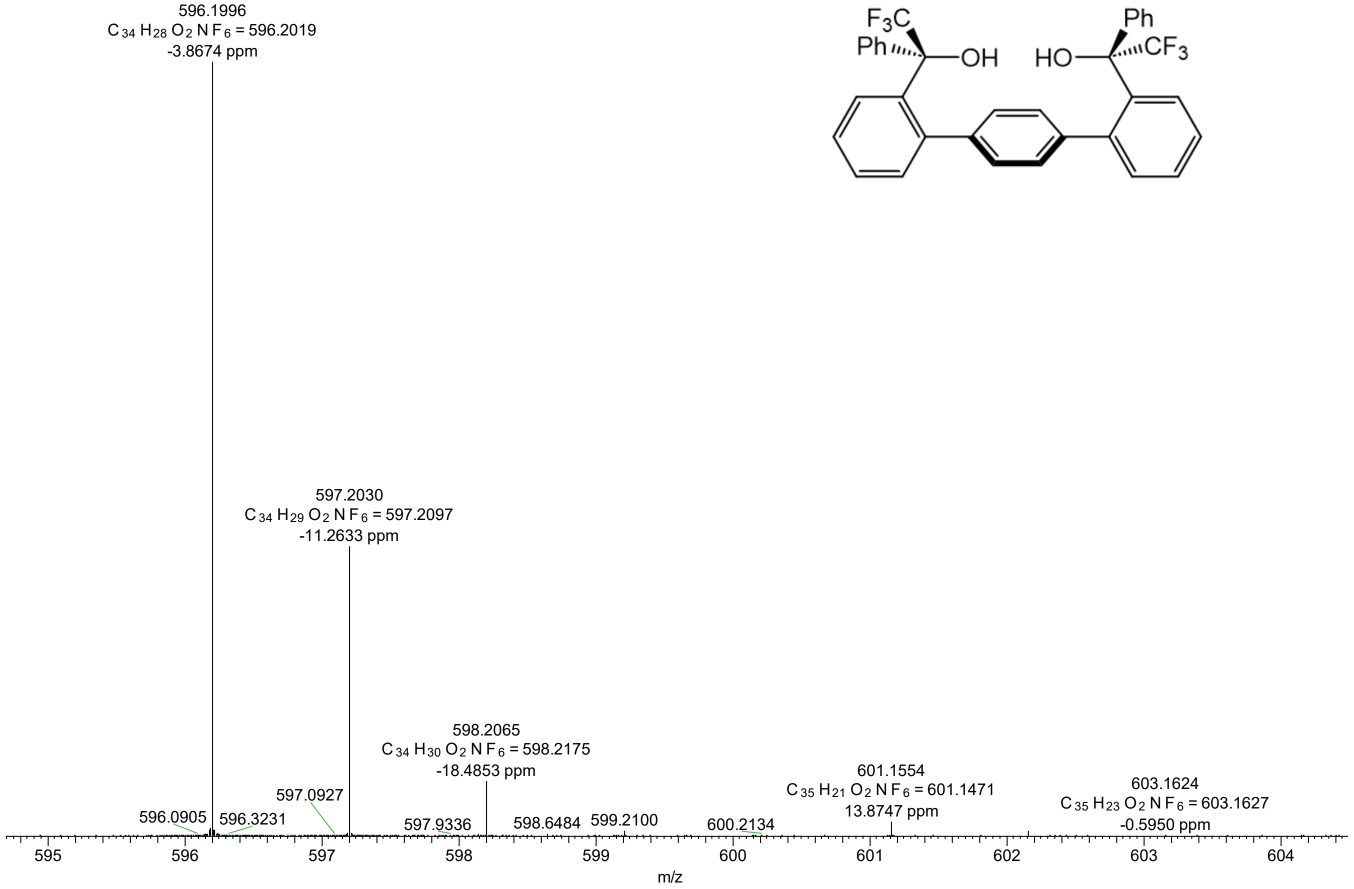

Figure S21. High resolution mass spectrum of *rac*-Lig^{2}H$_2$.

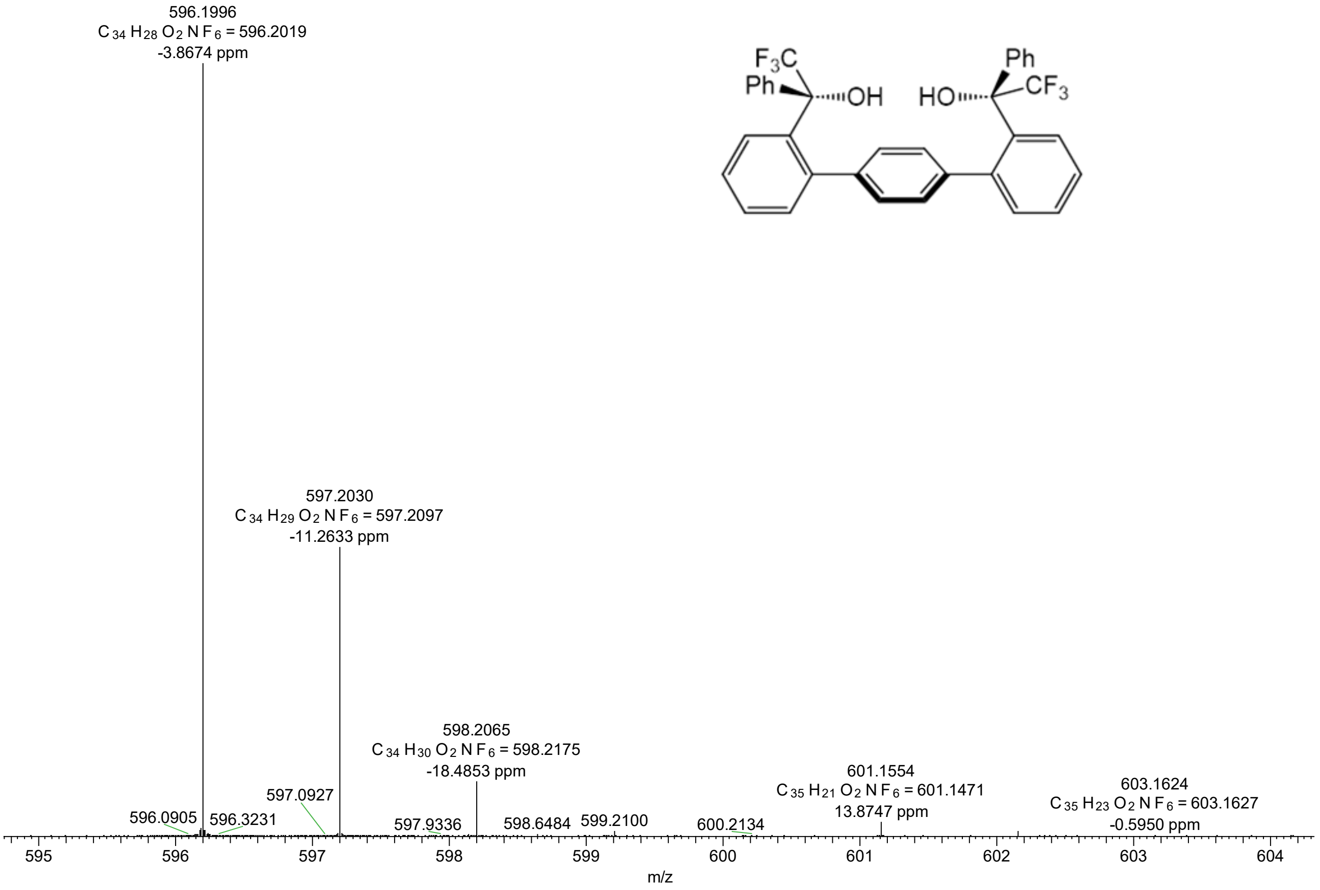

Figure S22. High resolution mass spectrum of *meso*-Lig^{2}H$_2$.

6. IR Spectra

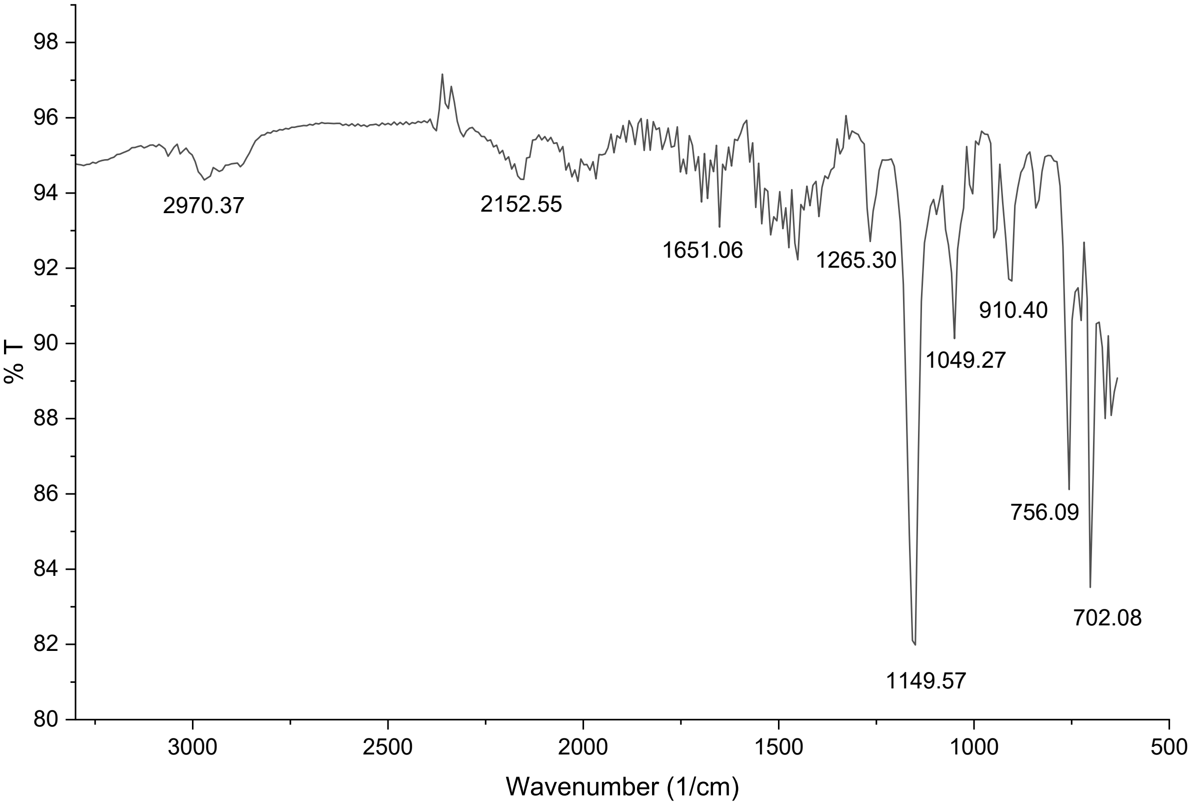

Figure S23. IR spectrum of Mg(*rac*-Lig^2)(THF)$_2$ (**1**).

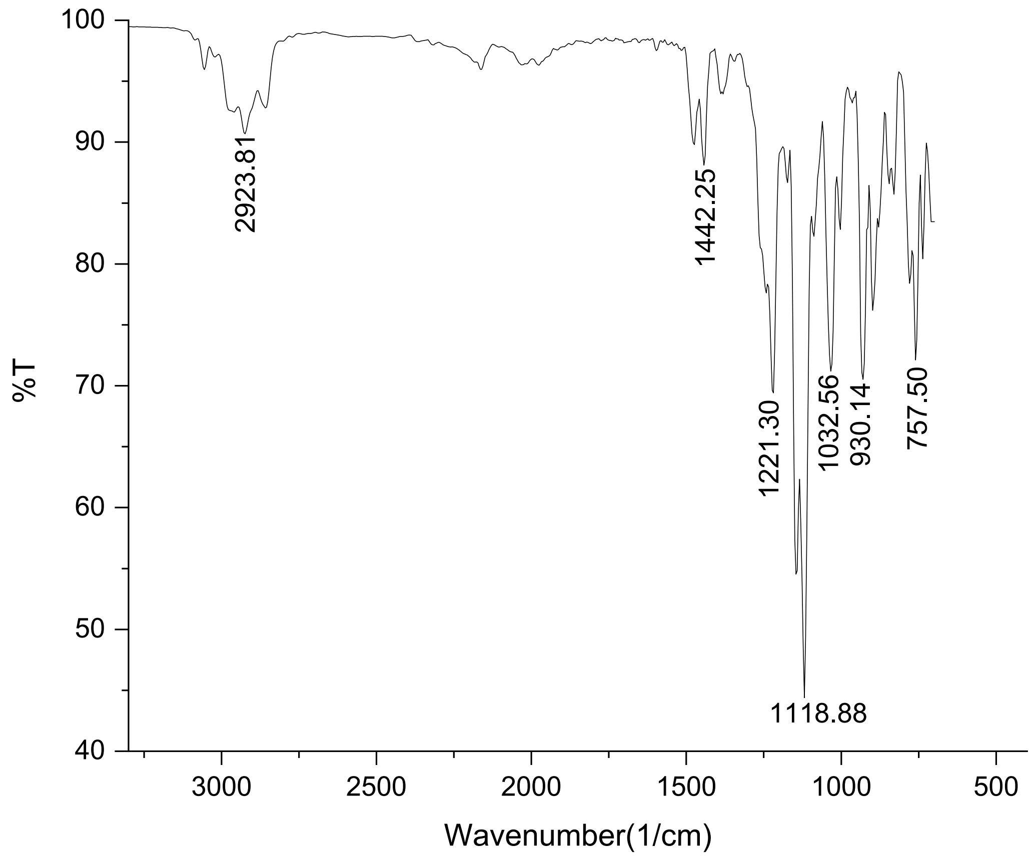

Figure S24. IR spectrum of Mg(*meso*-Lig^2)(THF)$_2$ (**2**).

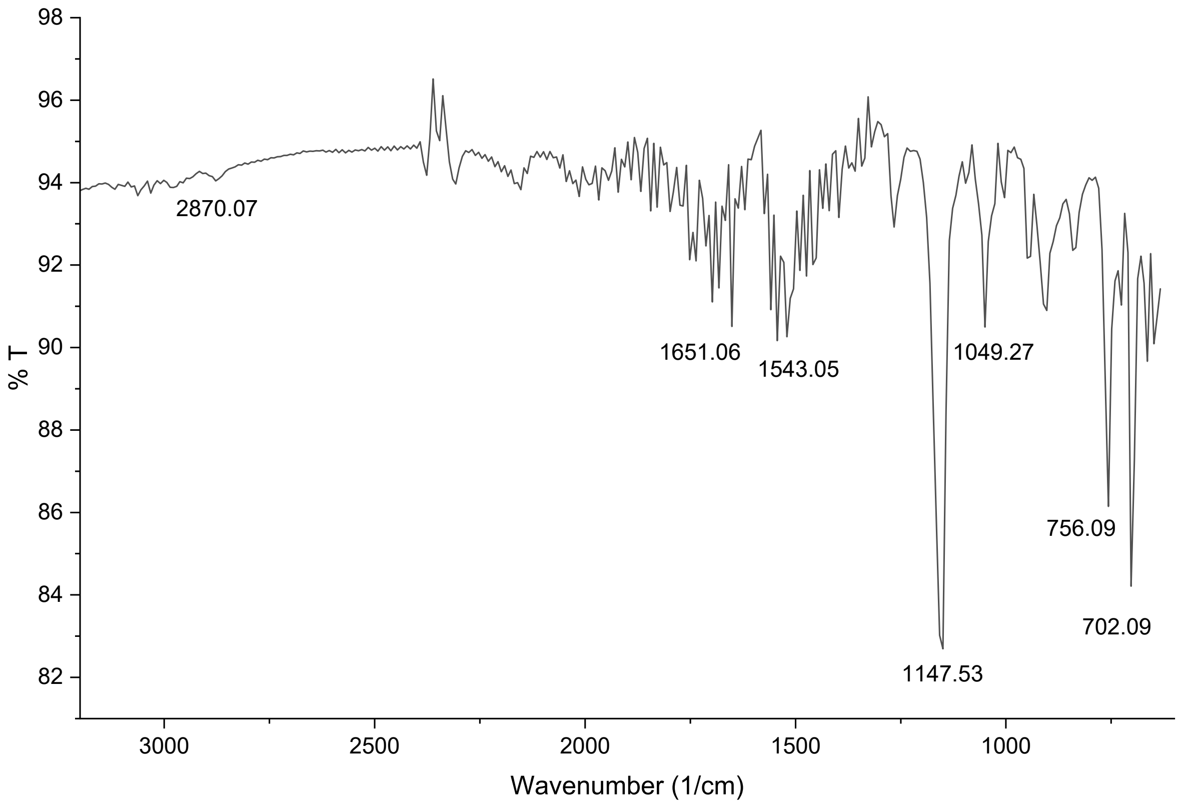

Figure S25. IR spectrum of Cr_2(*rac*-Lig^2)$_2$(THF)$_4$ (**4**).

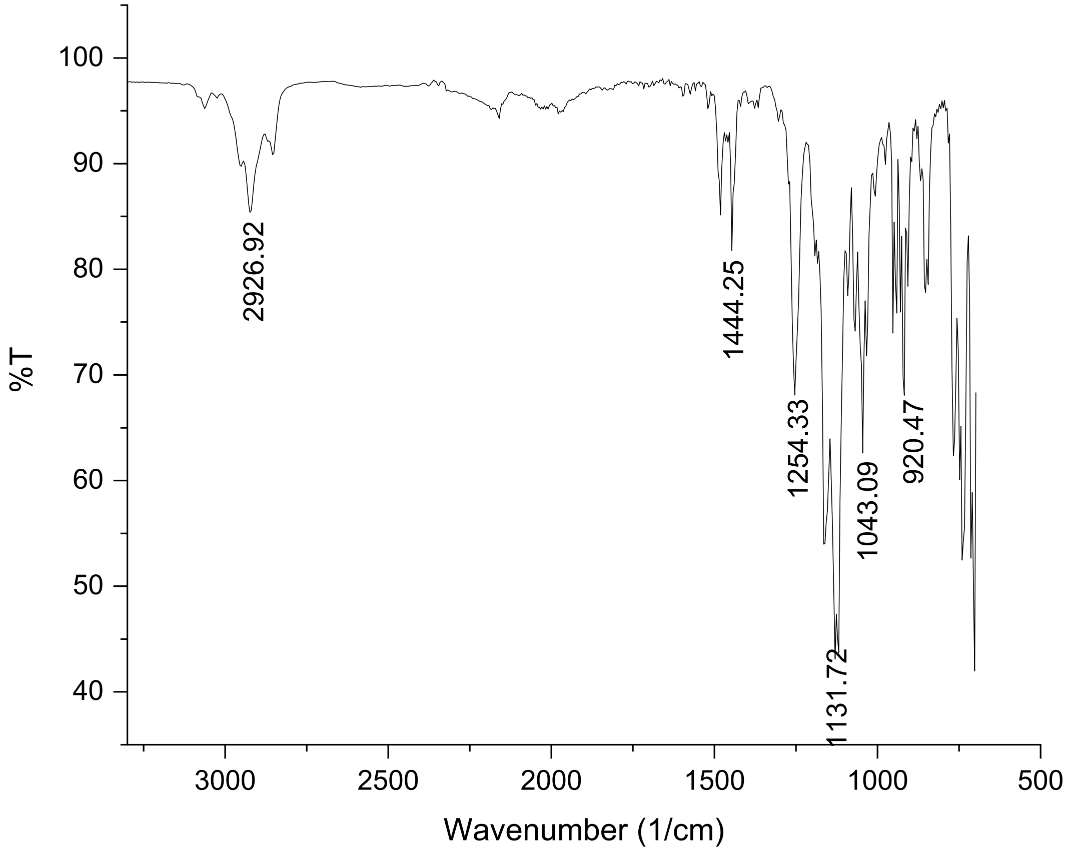

Figure S26. IR spectrum of Cr_2(*meso*-Lig^2)$_2$ (**3**).

7. Evans method formula and procedure

The Evans method was performed on all complexes using a Varian coaxial insert (purchased from Norell) and a standard NMR tube. The sample was carefully weighed, and a precise amount of the appropriate NMR solvent was added to afford solutions with known concentrations for the calculations. The solutions were added to the insert, and the insert was placed inside the outer NMR tube, which contained blank NMR solvent. NMR spectra were taken as indicated previously. The molar susceptibility χ_m of the compound was first calculated using Equation 1:[5]

$$\chi_m = \left[\frac{3\Delta v}{4\pi m v_0} + \chi_0\right] M \qquad (1)$$

where Δv is the peak separation in Hertz, m is the concentration of the solution in grams per milliliter, v_0 is the spectrometer operating frequency in Hertz, χ_0 is the molar susceptibility of the solvent (in cm^3/g), and M is the molar mass of the compound (g/mol). Molar susceptibilities (Pascal's constants) of the solvents used were obtained from *Journal of Chemical Education*.[6]

The solution state effective magnetic moment (μ_{eff}) was calculated using Equation 2:

$$\mu_{eff} = \sqrt{(2.383 \times 10^3)\,(\chi_m)} \qquad (2)$$

Two measurements were conducted for each compound, giving essentially the same value of the magnetic moment.

Table S3. Magnetic moments for complexes **3** and **4** using the Evans method.

**Complex 3**	μ_{obs} (μ_B)
1	3.8
2	3.7
**Complex 4**	μ_{obs} (μ_B)
1	4.3
2	4.4

8. Computational Details

Geometry optimizations were performed at the BP86/def2-SVP level of theory as implemented in Gaussian 16.[8] For the dimers we started with the crystallographic structure and modified one of the stereocenters (*i.e.* $\mathbf{3}_{meso}$ → $\mathbf{3}_{rac}$, and $\mathbf{4}_{rac}$ → $\mathbf{4}_{meso}$). Mono-chromium initial structures were made by pruning pieces from the dimer structures (*i.e.* $\mathbf{3}_{meso}$ → Cr(meso-Lig^2)(THF)$_2$ → Cr(meso-Lig^2)). For the free bis(alkoxide) ligand dianions, structures were built from scratch and pre-optimized with GOAT[9] and xTB,[10] and the lowest energy isomers after DFT refinement matched with the crystal structures. Density fitting and ultrafine grids were used, and all wavefunctions were confirmed to be stable.[11] Optimized structures were verified as minima through analysis of the harmonic frequencies.[12] Cartesian coordinates of all optimized structures may be found in the accompanying xyz file.

Gibbs free energies were estimated using standard approximations at the BP86/def2-SVP level of theory. Single point energy refinements were performed in ORCA 6.1.0 at the BP86-D4/def2-TZVP/SMD(THF), B3LYP-D4/def2-TZVP/SMD(THF), and ω-B97X-D4/def2-TZVP/SMD(THF)[13] levels of theory. Refined free energies were estimated using **Eq. 3**. B3LYP-D4/def2-TZVP/SMD(THF)//BP86/def2-SVP energies were reported in the manuscript, but all three sets of energies are included in this Supporting Information for completeness. While answers differed quantitatively from one functional to another, the chemical interpretation did not vary except in some cases of predicted spin states, where there is well-documented sensitivity based on the amount of exact exchange in the functional employed.[14] As a result, only B3LYP refined energies will be discussed. Full thermodynamics for each optimized species (and electronic energies for the fragment calculations) may be found in **Table S7**.

$$G_{TZ} = G_{DZ} - E_{DZ} + E_{TZ} \quad (3)$$

Optimization of Cr(*rac*-Lig^2) and Cr(*meso*-Lig^2) were performed initially. Due to the low coordination number and weak-field nature of our alkoxide ligands, only triplet and quintet states were considered. The quintet state is favored by each functional (including BP86 that is known to overstabilize low-spin states) and shows Mulliken spins of 3.8-4.1 at Cr, consistent with high-spin Cr(II). Addition of two THF molecules to form Cr(*rac*-Lig^2)(THF)$_2$ and Cr(*meso*-Lig^2)(THF)$_2$ is favored by 6.1 and 8.6 kcal/mol, respectively, and quintets are again favored by each functional. Optimized structures for all four quintet states are shown in **Figure S27**. It is noteworthy that all four structures show Cr-C_{arene} interactions as observed in the crystal structure of $\mathbf{3}_{meso}$.

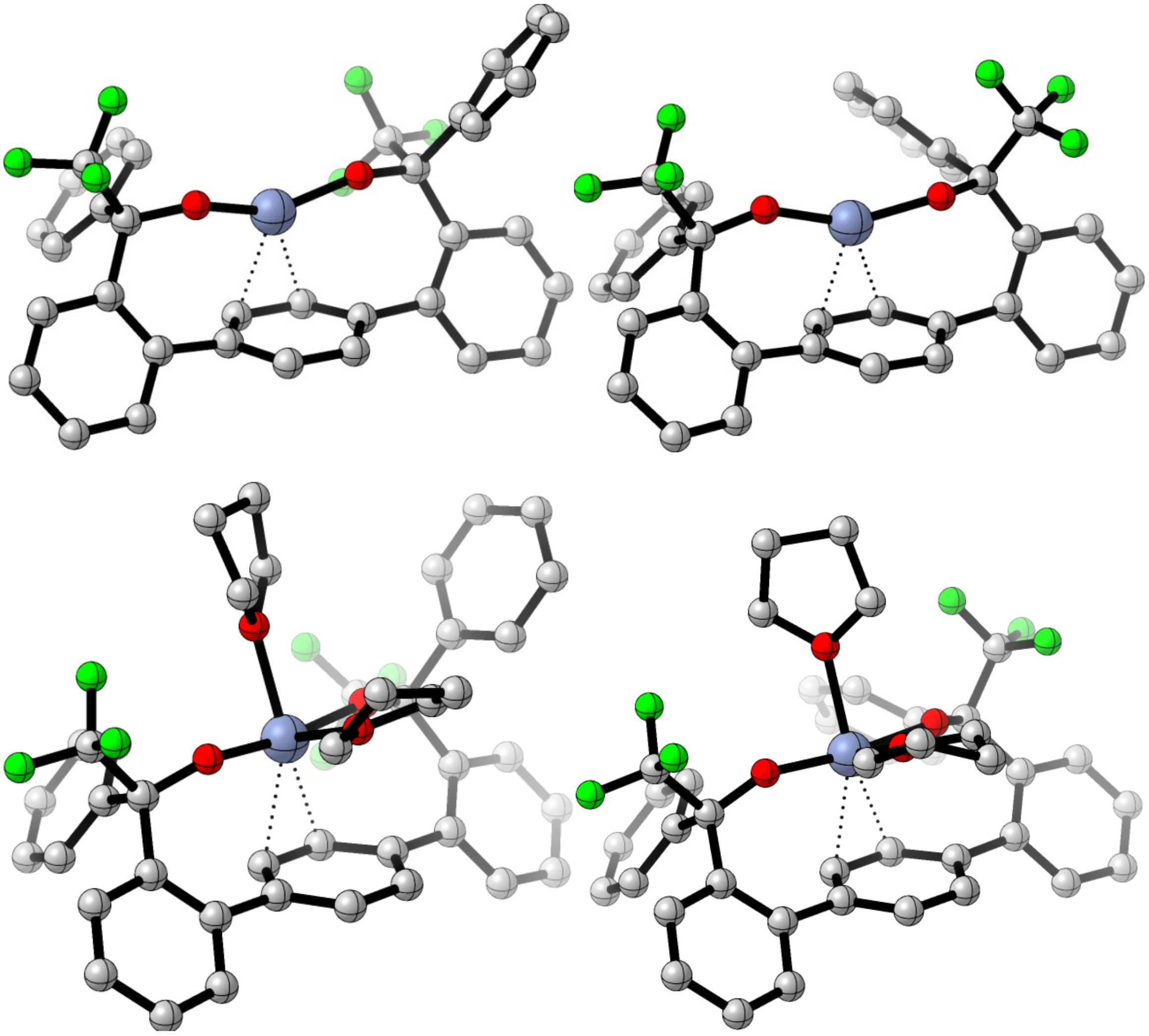

Figure S27. Optimized structures of Cr(*rac*-Lig^2) (top left), Cr(*meso*-Lig^2) (top right), Cr(*rac*-Lig^2)(THF)$_2$ (bottom left), Cr(*meso*-Lig^2)(THF)$_2$ (bottom right). Hydrogens omitted for clarity.

For the dimers, nonets were initially considered. However, based on the Evans' data in **Section 8**, we also optimized quintets (closest to experimental data) and the singlet state of crystallographically characterized species **3***meso*. Our rationale for only optimizing this singlet is that the Cr(II) centers are closest in this species, providing the best opportunity for magnetic coupling between the metal centers. As **Table S4** demonstrates, the nonet that corresponds to ferromagnetically coupled high-spin Cr(II) centers (based on Mulliken spins of 3.8-4.1 per chromium) and the singlet that correspond to antiferromagnetically coupled high-spin Cr(II) centers (based on Mulliken spins of 3.8-4.1 on one chromium and -3.8 to -4.1 on the other) is highly dependent on choice of functional, but all three functionals show these states to be nearly isoenergetic. In all cases the quintet states are overwhelmingly disfavored, regardless of functional. Thus, we focus on the nonet states in the manuscript but based on the magnetic data there may be

an equilibrium of the singlet and nonet states. Optimized structures of **3**$_{rac}$ and **3**$_{meso}$ are in the manuscript (**Figure 5**). Optimized structures of **4**$_{rac}$ and **4**$_{meso}$ may be found in **Figure S28**.

Table S4. Thermodynamics (in kcal/mol) of the various dimer spin states.

Cmpd	Spin State	$\Delta G_{BP86/TZ}$	$\Delta G_{B3LYP/TZ}$	$\Delta G_{wB97X/TZ}$
**3**$_{rac}$	quintet	+26.66	+38.02	+42.50
	nonet	0.00	0.00	0.00
**3**$_{meso}$	singlet	-1.06	+2.35	+4.31
	quintet	+29.00	+40.45	+45.27
	nonet	0.00	0.00	0.00
**4**$_{rac}$	quintet	+50.65	+54.89	+58.37
	nonet	0.00	0.00	0.00
**4**$_{meso}$	quintet	+51.78	+55.82	+59.09
	nonet	0.00	0.00	0.00

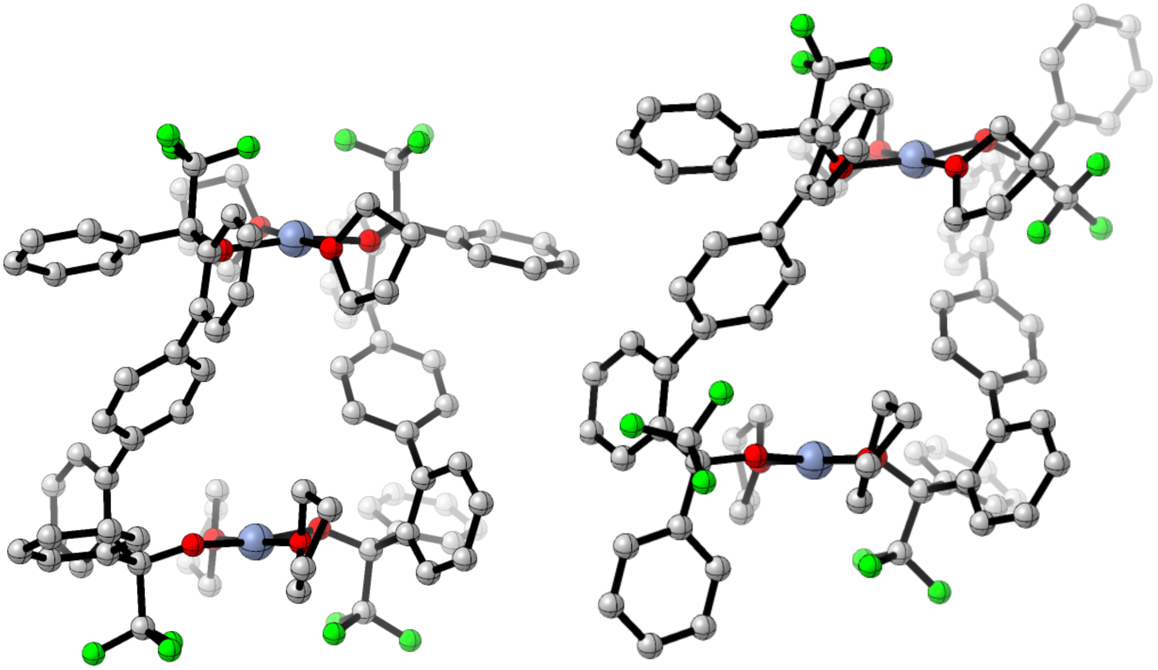

Figure S28. Optimized structures of Cr_2(*rac*-Lig^2)$_2$(THF)$_4$ (left) and Cr_2(*meso*-Lig^2)$_2$(THF)$_4$ (right). Hydrogens omitted for clarity.

Table S5. Selected bond lengths (Å) from the optimized structures of the dimers.

Bond	**3**$_{rac}$	**3**$_{meso}$	**4**$_{rac}$	**4**$_{meso}$
Cr-Cr	2.907	2.970	7.546	7.698
Cr-O$_{bridging}$	2.102<br>2.101<br>2.089	2.102<br>2.102<br>2.063	-	-

	2.088	2.063		
Cr-$O_{terminal}$	1.922<br>1.922	1.899<br>1.899	1.956<br>1.953<br>1.950<br>1.947	1.976<br>1.953<br>1.937<br>1.922
Cr-O_{THF}	-	-	2.110<br>2.101<br>2.092<br>2.089	2.096<br>2.092<br>2.084<br>2.083
Cr-C_{arene}	2.732<br>2.723<br>2.534<br>2.534	2.585<br>2.584<br>2.462<br>2.462	-	-

To understand the thermodynamic preference for these dimers as a function of the ligand stereoisomer, we took inspiration from the Distortion/Interaction-Activation Strain model,[15] though we did not employ energy decomposition analyses to further break down these energies. For **3***rac* and **3***meso*, the data is fully explained in the paper. For **4***rac* and **4***meso*, we need to break down the pre-organization energy into the three components discussed in the manuscript: (i) distorting the bis(alkoxides) to their final conformations and bringing them together, (ii) distorting the THFs to their final conformations and bringing them together, and (iii) letting these two ligand fragments interact. As **Table S6** demonstrates, ΔE_{ii} and ΔE_{iii} are almost identical for the two dimer structures. The THFs are stabilized slightly by dispersion interactions, while putting all those oxygens in proximity comes with an energetic penalty that is nearly identical for the two structures given the similar Cr-O bond lengths (**Table S5**). Thus, the origin of the difference is in ΔE_i. Moreover, we can further decompose this into the distortion to the individual conformations (~ +2.7 kcal/mol per *rac*-Lig^2 *vs.* ~ +8.7 kcal/mol per *meso*-Lig^2) that shows it is the conformational change that is the origin of this effect, as the electrostatic penalty of bringing those distorted ligands together is nearly equal at +37.54 and +37.32 kcal/mol for **4***rac* and **4***meso*, respectively.

Table S6. Thermodynamics (kcal/mol) breaking down the pre-organization energy of the ligands in **4***rac* and **4***meso* into contributions from (i) bis(alkoxides), (ii) THFs, and (iii) letting each of those interact.

Reaction	ΔE_i	ΔE_{ii}	ΔE_{iii}	$\Delta E_{pre\text{-}org}$
2 Cr^{2+} + 2 (*rac*-Lig2)$^{2-}$ + 4 THF → **4***rac*	+29.47	-0.72	+43.50	+72.25
2 Cr^{2+} + 2 (*meso*-Lig2)$^{2-}$ + 4 THF → **4***meso*	+41.10	-0.54	+43.80	+84.36

Table S7. Thermodynamics (E_h) for all fully optimized species and electronic energies (only) for fragments. BP = BP86, B3 = B3LYP, wB = ω-B97X, DZ = def2-SVP, and TZ = def2-TZVP.

Species	$E_{BP/DZ}$	$G_{BP/DZ}$	$E_{BP/TZ}$	$E_{B3/TZ}$	$E_{wB/TZ}$
Cr(*rac*-Lig^2)	-3101.417979	-3101.027739	-3104.068357	-3103.935717	-3104.429135

S=2					
Cr(*rac*-Lig^2) *S*=1	-3101.423926	-3101.029618	-3104.072387	-3103.909736	-3104.406588
Cr(*meso*-Lig^2) *S*=2	-3101.416442	-3101.025330	-3104.072286	-3103.938280	-3104.432682
Cr(*meso*-Lig^2) *S*=1	-3101.407609	-3101.014937	-3104.056273	-3103.896604	-3104.388236
Cr(*rac*-Lig^2)$(THF)_2$ *S*=2	-3566.000300	-3565.395026	-3569.225883	-3569.115844	-3569.779727
Cr(*rac*-Lig^2)$(THF)_2$ *S*=1	-3565.964297	-3565.359005	-3569.185502	-3569.065395	-3569.725584
Cr(*meso*-Lig^2)$(THF)_2$ *S*=2	-3566.000109	-3565.395250	-3569.230754	-3569.121101	-3569.785581
Cr(*meso*-Lig^2)$(THF)_2$ *S*=1	-3565.962504	-3565.356967	-3569.191017	-3569.074059	-3569.735128
$\mathbf{3}_{rac}$ *S*=4	-6202.860583	-6202.044490	-6208.205190	-6207.952815	-6208.941116
$\mathbf{3}_{rac}$ *S*=2	-6202.822114	-6202.004349	-6208.164379	-6207.893901	-6208.875062
$\mathbf{3}_{meso}$ *S*=4	-6202.873047	-6202.056128	-6208.179493	-6207.906370	-6208.886114
$\mathbf{3}_{meso}$ *S*=2	-6202.833599	-6202.014961	-6208.144989	-6207.883356	-6208.865240
$\mathbf{3}_{meso}$ *S*=0	-6202.880752	-6202.061165	-6208.228346	-6207.968030	-6208.952346
$\mathbf{4}_{rac}$ *S*=4	-7132.019041	-7130.782890	-7138.514817	-7138.300761	-7139.615585
$\mathbf{4}_{rac}$ *S*=2	-7131.966989	-7130.730852	-7138.433127	-7138.213775	-7139.523946
$\mathbf{4}_{meso}$ *S*=4	-7132.006761	-7130.773716	-7138.409661	-7138.189414	-7139.499813
$\mathbf{4}_{meso}$ *S*=2	-7131.957065	-7130.723369	-7138.407490	-7138.189949	-7139.501648
(*rac*-$Lig^2)^{2-}$ *S*=0	-2056.864661	-2056.480604	-2059.543609	-2059.543166	-2060.087434
(*meso*-$Lig^2)^{2-}$ *S*=0	-2056.868823	-2056.483368	-2059.543864	-2059.542979	-2060.087035
THF *S*=0	-232.272992	-232.188699	-232.554860	-232.562007	-232.646810
Cr^{2+} *S*=2	-1043.520846	-1043.537949	-1044.161280	-1044.053912	-1044.005642
(*rac*-$Lig^2)_2^{4-}$ @ $\mathbf{3}_{rac}$ *S*=0	-	-	-4118.923303	-4118.913746	-4119.994383
(*meso*-$Lig^2)_2^{4-}$ @ $\mathbf{3}_{meso}$ *S*=0	-	-	-4118.916176	-4118.908015	-4119.989802
(*rac*-$Lig^2)_2^{4-}$ @ $\mathbf{4}_{rac}$ *S*=0	-	-	-5049.175921	-5049.199553	-5050.620867
$(THF)_4^0$ @ $\mathbf{4}_{rac}$ *S*=0	-	-	-930.219182	-930.248886	-930.586539
(*rac*-$Lig^2)_2(THF)_4^{4-}$ @ $\mathbf{4}_{rac}$ *S*=0	-	-	-6208.086045	-6207.845173	-6208.823720
(*meso*-$Lig^2)_2^{4-}$ @ $\mathbf{4}_{meso}$ *S*=0	-	-	-5049.195259	-5049.219226	-5050.640331
$(THF)_4^0$ @ $\mathbf{4}_{meso}$ *S*=0	-	-	-930.219440	-930.249182	-930.586586

(*meso*-$Lig^2)_2(THF)_4^{4-}$ @ $\mathbf{4}_{meso}$ S=0	-	-	-6208.107239	-6207.868793	-6208.847198

9. References

1. a) X. Feng, W. Pisula and K. Mullen, *J. Am. Chem. Soc.*, **2007**, *129*, 14116-14117. b) A. Velian, S. Lin, A. J. M. Miller, M. W. Day and T. Agapie, *J. Am. Chem. Soc.*, **2010**, *132*, 6296– 6297.

2. Sheldrick, G., SHELXT - Integrated space-group and crystal-structure determination. *Acta Cryst. Section A* **2015**, *71*, 3-8.

3. Sheldrick, G., Crystal structure refinement with SHELXL. *Acta Cryst. Section C* **2015,** *71*, 3-8.

4. Dolomanov, O. V.; Bourhis, L. J.; Gildea, R. J.; Howard, J. A. K.; Puschmann, H., OLEX2: a complete structure solution, refinement and analysis program. *J. Appl. Crystallogr.* **2009**, *42*, 339-341.

5. Evans, D. F. *J. Chem. Soc*. **1959**, 2003-2005.

6. Bain, G. A.; Berry, J. F. *J. Chem. Ed*. **2008**, *85*, 532-536.

7. Bradley, D. C.; Hursthouse, M. B.; Newing, C. W.; Welch, A. *J. Chem. Soc., Chem. Commun.,* **1972***,* 567-568.

8. Most of these methods were already cited in the manuscript – please refer to references 60-76. Only papers/software not cited there are included in the SI references.

9. De Souza, B. GOAT: A Global Optimization Algorithm for Molecules and Atomic Clusters. *Angew. Chem. Int. Ed.* **2025**, *64*, e202500393.

10. Bannwarth, C.; Ehlert, S.; Grimme, S. GFN2-xTB – An Accurate and Broadly Parametrized Self-Consistent Tight-Binding Quantum Chemical Method with Multipole Electrostatics and Density-Dependent Dispersion Contributions. *J. Chem. Theory Comput.* **2019**, *15*, 1652-1671.

11. Bauernschmitt, R.; Ahlrichs, R. Stability analysis for solutions of the closed shell Kohn-Sham equation. *J. Chem. Phys.* **1996**, *104*, 9047-9052.

12. Schlegel, H. B. Geometry optimization. *WIREs Comput. Mol. Sci.* **2011**, *1*, 790-809.

13. Chai, J.-D.; Head-Gordon, M. Long-range corrected hybrid density functionals with damped atom-atom dispersion corrections. *Phys. Chem. Chem. Phys.* **2008**, *10*, 6615-6620.

14. Swart, M.; Gruden, M. Spinning around in Transition-Metal Chemistry. *Acc. Chem. Res.* **2016**, *49*, 2690-2697.

15. Bickelhaupt, F. M.; Houk, K. N. Analyzing Reaction Rates with the Distortion/Interaction-Activation Strain Model. *Angew. Chem. Int. Ed.* **2017**, *56*, 10070-10086.
